# Identification of FAM173B as a protein methyltransferase promoting chronic pain

**DOI:** 10.1371/journal.pbio.2003452

**Published:** 2018-02-14

**Authors:** Hanneke L. D. M. Willemen, Annemieke Kavelaars, Judith Prado, Mirjam Maas, Sabine Versteeg, Lara J. J. Nellissen, Jeshua Tromp, Rafael Gonzalez Cano, Wenjun Zhou, Magnus E. Jakobsson, Jędrzej Małecki, George Posthuma, Abdella M. Habib, Cobi J. Heijnen, Pål Ø. Falnes, Niels Eijkelkamp

**Affiliations:** 1 Laboratory of Neuroimmunology and Developmental Origins of Disease (NIDOD), University Medical Center Utrecht, Utrecht University, Utrecht, the Netherlands; 2 Laboratory of Neuroimmunology, University of Texas M.D. Anderson Cancer Center, Houston, Texas, United States of America; 3 Laboratory of Translational Immunology, University Medical Center Utrecht, Utrecht University, Utrecht, the Netherlands; 4 Department of Pharmacology and Institute of Neuroscience, University of Granada, Granada, Spain; 5 Department of Biosciences, Faculty of Mathematics and Natural Sciences, University of Oslo, Oslo, Norway; 6 Department of Cell Biology and Institute of Biomembranes, Center for Molecular Medicine, University Medical Center Utrecht, Utrecht University, Utrecht, the Netherlands; 7 Molecular Nociception Group, Wolfson Institute for Biomedical Research, University College London, London, United Kingdom; 8 College of Medicine, Member of Qatar Health, Qatar University, Doha, Qatar; University of Heidelberg, Germany

## Abstract

Chronic pain is a debilitating problem, and insights in the neurobiology of chronic pain are needed for the development of novel pain therapies. A genome-wide association study implicated the 5p15.2 region in chronic widespread pain. This region includes the coding region for FAM173B, a functionally uncharacterized protein. We demonstrate here that FAM173B is a mitochondrial lysine methyltransferase that promotes chronic pain. Knockdown and sensory neuron overexpression strategies showed that FAM173B is involved in persistent inflammatory and neuropathic pain via a pathway dependent on its methyltransferase activity. FAM173B methyltransferase activity in sensory neurons hyperpolarized mitochondria and promoted macrophage/microglia activation through a reactive oxygen species–dependent pathway. In summary, we uncover a role for methyltransferase activity of FAM173B in the neurobiology of pain. These results also highlight FAM173B methyltransferase activity as a potential therapeutic target to treat debilitating chronic pain conditions.

## Author summary

Pain is an evolutionarily conserved physiological phenomenon necessary for survival. Yet, pain can become pathological when it occurs independently of noxious stimuli. The molecular mechanisms of pathological pain are still poorly understood, limiting the development of highly needed novel analgesics. Recently, genetic variations in the genomic region encoding FAM173B—a functionally uncharacterized protein—have been linked to chronic pain in humans. In this study, we identify the role and function of FAM173B in the development of pathological pain. We used genetic, biochemical, and behavioral approaches in mice to show that FAM173B is a mitochondrial lysine methyltransferase—a protein that transfers methyl group to donor proteins. By genetically silencing or overexpressing FAM173B in sensory neurons, we showed that FAM173B methyltransferase activity promotes the development of chronic pain. In addition, we discovered that FAM173B methyltransferase activity in the mitochondria of sensory neurons promotes chronic pain via a pathway that depends on the production of reactive oxygen species and on the engagement of spinal cord microglia—engulfing cells of the central nervous system. These data point to an essential role of FAM173B in the regulation of pathological pain.

## Introduction

Chronic pain is a major clinical problem and affects approximately 20% of the population [1–3]. Inflammation, tissue, and nerve damage induce long-lasting changes in the nociceptive circuitry, causing pain and exaggerated responses to noxious and innocuous stimuli [4, 5]. Although many efforts have been undertaken to elucidate the molecular pathways driving chronic pain, a complete understanding of the mechanisms leading to chronic pain is missing, hampering the development of highly needed therapeutic approaches to treat debilitating pain conditions.

At the mechanistic level, the activation of spinal cord glial cells is thought to drive persistent pain. In various rodent models of chronic pain, including neuropathic and persistent inflammatory pain, spinal cord microglia have an activated phenotype and produce inflammatory mediators that trigger or maintain the long-lasting changes in nociceptive circuitry, thereby contributing to persistent pain [6–10]. Many efforts have been undertaken to elucidate how peripheral sensory neurons drive the engagement of these glial cells in chronic pain conditions. Sensory neurons engage spinal glial cells through the release of soluble factors [6, 11, 12]. However, the intracellular pathways in sensory neurons upstream of the release of glia-activating factors are still unknown.

Another driving force of pathological pain is the formation of reactive oxygen species (ROS) [13]. ROS are derived from electrons leaking from the mitochondrial electron transport chain and can initiate proinflammatory cascades and activate microglia in the central nervous system [14]. Importantly, increased ROS levels in the dorsal root ganglia (DRG) and/or spinal cord contribute to chronic pain development in several rodent models [13, 15–18], and altered ROS levels are associated with chronic pain development in humans [19–21].

Further understanding of the mechanism that drives pathological pain is needed. The identification of novel “pain genes” that lie at the root of the transition from acute to persistent pain, possibly through glial cell engagement and ROS formation, aids in this understanding and could identify highly needed novel targets for therapeutic pain interventions. Several genome-wide association studies (GWAS) in humans have offered a glimpse of the genetic contributions to pain syndromes. Nevertheless, very few have pinpointed new pain genes that provided novel insights in pain neurobiology. Recently, specific single nucleotide polymorphisms (SNPs) have been identified in patients with chronic widespread pain in a large-scale GWAS [22]. Two top intronic SNPs on chromosome 5p15.2 were shown to be associated with a 30% higher risk of developing chronic widespread pain. This genomic region encodes Chaperonin Containing TCP1 Subunit 5 (CCT5) and the hitherto functionally uncharacterized FAM173B protein, indicating potential novel pain genes. The 2 top SNPs found in the GWAS are linked to a nonsynonymous SNP (rs2438652) in the *FAM173B* gene and to 1 intronic SNP in *FAM173B* (rs2445871) that has a predicted effect on FAM173B expression levels [22, 23]. However, the molecular function of FAM173B and its potential role in the neurobiology of chronic pain have not been revealed. Here, we identify FAM173B as a lysine-specific protein methyltransferase that resides in the mitochondrial cristae and show that neuronal FAM173B methyltransferase activity controls the development of chronic pain through an ROS-dependent pathway resulting in the activation of glial cells.

## Results

### FAM173B promotes chronic pain development in vivo

**Fig 1:**
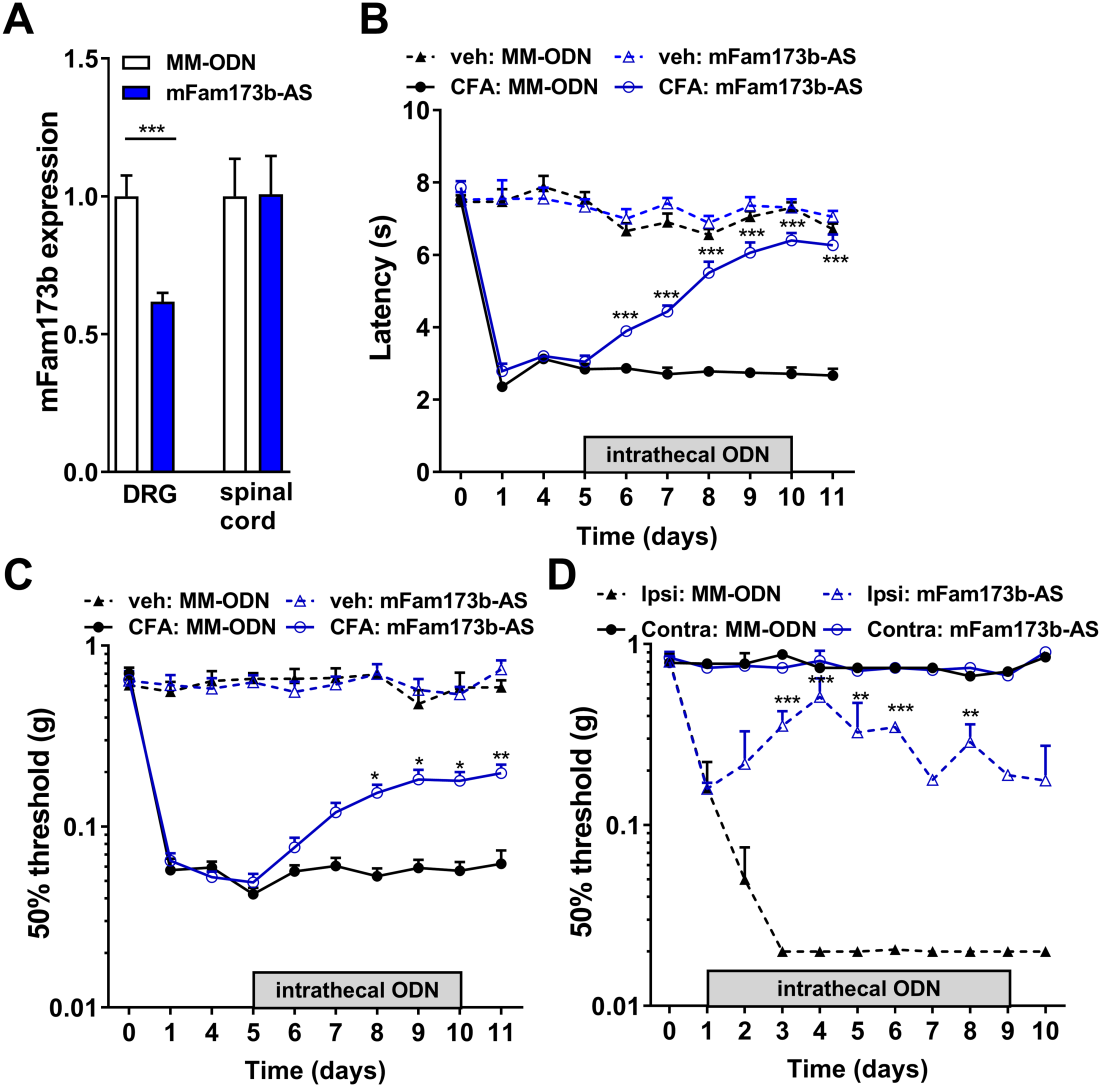
FAM173B knockdown abrogated persistent inflammatory and neuropathic pain. (A) Intrathecal *mFam173b* antisense ODN (*mFam173b-AS*) injection at day 5, 6, 7, 9, and 10 after intraplantar injection of CFA reduces *mFam173b* mRNA expression (corrected for housekeeping genes *GAPDH* and *HPRT*) in the DRG 24 hours after the last *mFam173b-AS* injection (*n* = 8 mice). (B–D) Time course of (B) thermal and (C, D) mechanical hyperalgesia following (B, C) intraplantar injection of CFA (*n* = 8 mice), veh (*n* = 4 mice), or (D) after SNI (*n* = 4 mice). Mice received intrathecal injections of *mFam173b-AS* or *MM-ODN* at days 5, 6, 7, 9, and 10 during inflammatory pain or day 1–9 after SNI. Data are represented as mean ± SEM. * = *P* < 0.05; ** = *P* < 0.01; *** = *P* < 0.001. Statistical analyses were performed by unpaired two-tailed *t* tests (A) or by two-way repeated measures ANOVA followed by a post-hoc Holm-Sidak multiple comparison test (B–D). Underlying data can be found in S1 Data. CFA, complete Freund’s adjuvant; contra, contralateral; DRG, dorsal root ganglia; GAPDH, glyceraldehyde 3-phosphate dehydrogenase; HPRT, Hypoxanthine Phosphoribosyltransferase 1; ipsi, ipsilateral; MM-ODN, mismatch ODN; ODN, oligodeoxynucleotide; SEM, standard error of the mean; SNI, spared nerve injury; veh, vehicle.

To determine whether FAM173B is involved in chronic pain, we down-regulated Fam173b expression in vivo by lumbar intrathecal injections of a nuclease-resistant antisense oligodeoxynucleotide (ODN), a method that has been shown to reduce mRNA expression and protein translation [24]. We injected mouse *Fam173b* antisense ODN (*mFam173b-AS*) intrathecal into the lumbar enlargement because, through this application route, antisense ODNs mainly target the lumbar DRGs [25–28]. Five daily intrathecal injections of *mFam173b-AS* reduced *mFam173b* mRNA expression in vivo in lumbar DRG in the complete Freund’s adjuvant (CFA) model of persistent inflammatory pain [29] and in vehicle-treated mice (S1B Fig), without affecting spinal cord *mFam173b* mRNA expression (Fig 1A). Intrathecal injection of a fluorescently labeled *mFam173b-AS* targeted almost all sensory neurons and some other cells, including ionized calcium binding adaptor molecule 1 (Iba1) and glial fibrillary acidic protein (GFAP)-positive cells in the DRG (S1A Fig). Intrathecal administration of *mFam173b-AS* at day 5 until 10 in the CFA model of persistent inflammatory pain abrogated thermal and mechanical hyperalgesia (Fig 1B/1C). These results were confirmed by using another *mFam173b-AS* targeted to a different region of *mFam173b* mRNA (S1B–S1D Fig), indicating the ODN-induced effects are likely not due to off-target effects. Intrathecal injections of *mFam173b-AS* from day 1 until 9 also attenuated the development of neuropathic pain in the spared nerve injury model [30]. Mechanical thresholds in the contralateral paw were not affected by *mFam173b-AS* treatment (Fig 1D).

**Fig 2:**
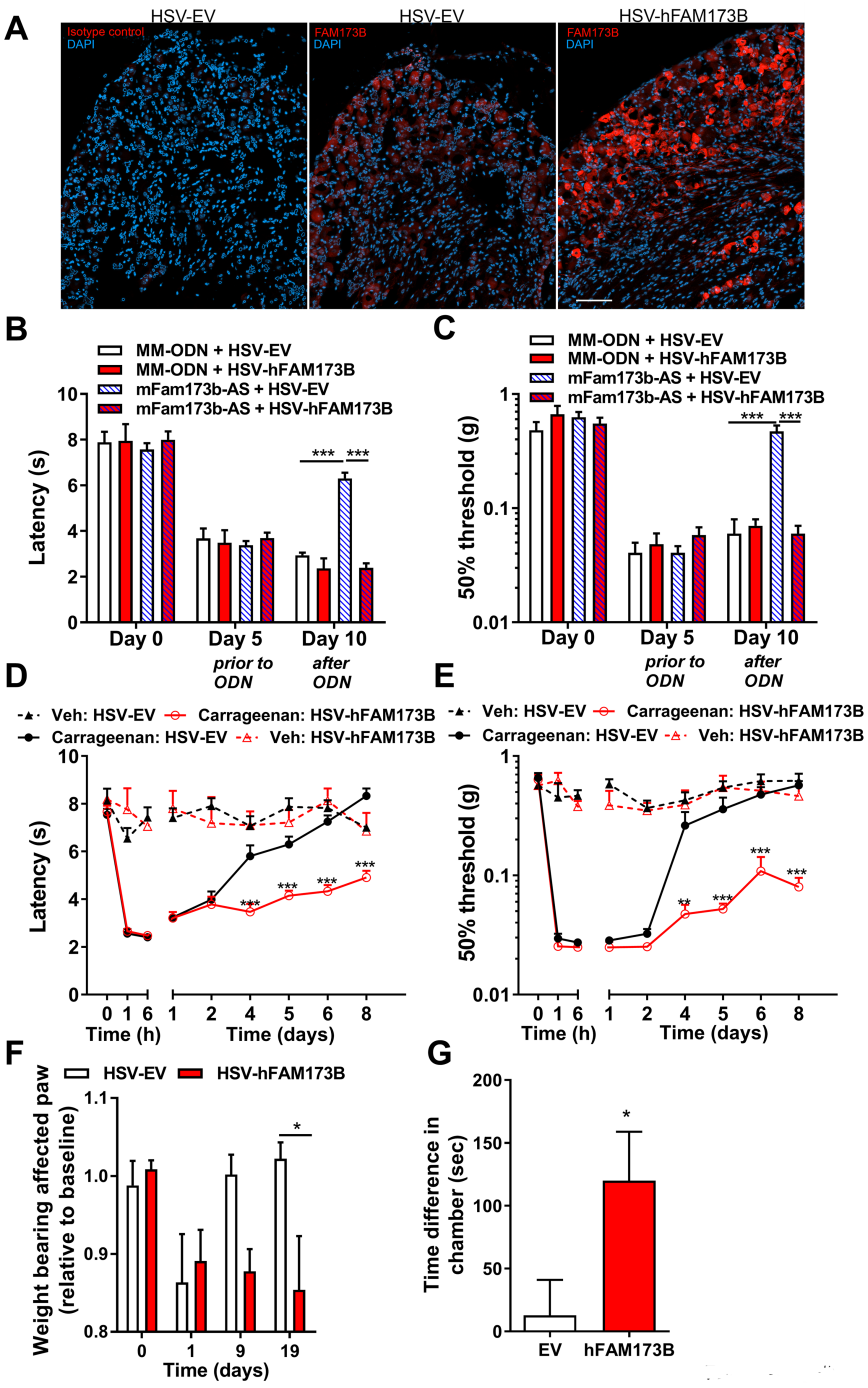
Sensory neuron FAM173B promotes chronic pain. (A) Intraplantar injections with HSV amplicons (day 1 and 3) encoding either for hFAM173B-GFP or GFP alone (HSV-EV) induced hFAM173B expression selectively in sensory neurons at day 4. Left panel depicts rabbit IgG control. Nuclei are visualized with DAPI (blue). Scale bar is 50 μm. For larger magnification of FAM173B staining of the DRG after HSV-hFAM173b, see S1 Fig. (B, C) Intraplantar HSV-hFAM173B injection at day 5 and 7 rescued *mFam173b-AS*–mediated attenuation of (B) thermal and (C) mechanical hypersensitivity in the CFA model of persistent inflammatory pain. Mice received intrathecal ODN at day 5, 6, 7, 9, and 10 after CFA (*mFam173b-AS n* = 8; *MM-ODN n* = 4 mice). (D, E) Intraplantar HSV-hFAM173B injections at 3 and 1 day prior to intraplantar carrageenan injection prolonged transient inflammatory (D) thermal and (E) mechanical hypersensitivity (carrageenan: *n* = 10, vehicle: *n* = 6 mice). (F) Intraplantar HSV-hFAM173B injections at 3 and 1 day prior to a unilateral intraplantar carrageenan injection reduced weight bearing of the affected paw that persisted at least until day 19 in HSV-FAM173B–injected but not HSV-EV–injected mice (*n* = 6 mice). (G) Ongoing spontaneous pain measured with gabapentin-induced place preference in HSV-hFAM173B– but not HSV-EV–treated mice 1 month after intraplantar carrageenan injection (*n* = 6 mice). Data are represented as mean ± SEM. * = *P* < 0.05; ** = *P* < 0.01; *** = *P* < 0.001. Statistical analyses were performed by unpaired two-tailed *t* tests (G) or by two-way repeated measures ANOVA followed by a post-hoc Holm-Sidak multiple comparison test (B–F). Underlying data can be found in S1 Data. CFA, complete Freund’s adjuvant; DAPI, 4′,6-diamidino-2-phenylindole; DRG, dorsal root ganglia; GFP, green fluorescent protein; HSV, herpes simplex virus; HSV-EV, HSV empty vector; IgG, immunoglobulin G; MM-ODN, mismatch ODN; ODN, oligodeoxynucleotide; SEM, standard error of the mean.

To test if FAM173B in sensory neurons is central to the inhibitory effect of intrathecal *mFam173b-AS* on chronic inflammatory pain, we performed a rescue experiment. We expressed human FAM173B (hFAM173B) in sensory neurons in vivo using herpes simplex virus (HSV)-mediated gene transfer in mice treated intrathecally with *mFam173b-AS* that does not recognize human *FAM173B* mRNA. HSV selectively infects primary sensory neurons, and intraplantar or intrathecal HSV amplicons encoding for hFAM173B and green fluorescent protein (GFP) transferred GFP (S1E Fig) and hFAM173B into sensory neurons in the DRG (Fig 2A and S1F Fig) but not to other cells in the DRG such as F4/80-positive macrophages (S1F Fig).

Intraplantar HSV-hFAM173B injections induced protein expression of hFAM173B detected by western blot in the lumbar DRG (S1G Fig). Furthermore, GFP was detected in peripherin-positive sciatic nerve fibers and peripherin-positive nerve endings in the skin of the injected hind paw, indicating gene transfer to sensory neurons (S1H/S1I Fig). This sensory neuron selective expression of proteins using HSV is consistent with previous literature [27, 31, 32]. Intraplantar (to target sensory neurons innervating the hind paw) (Fig 2B/2C) or intrathecal (S1J/S1K Fig) administration of HSV-hFAM173B completely prevented the *mFam173b-AS*–mediated attenuation of persistent thermal and mechanical hypersensitivity in the CFA model, indicating that sensory neuron FAM173B is required for persistent inflammatory pain.

Next, we tested whether increasing sensory neuron hFAM173B is sufficient to promote the transition of transient inflammatory pain into persistent pain. Intraplantar injection of 5 μl of 1% carrageenan induced transient hyperalgesia [27, 33] that resolved within 4 to 6 days (Fig 2D/2E). Intraplantar (Fig 2D/2E) or intrathecal (S1L/S1M Fig) administration of HSV-hFAM173B prior to the induction of transient inflammatory pain markedly prolonged carrageenan-evoked thermal and mechanical hyperalgesia as compared to mice treated with control HSV empty vector (HSV-EV). A single carrageenan injection in one paw induces a reduction in weight bearing of the affected paw that normalizes within 9 days. However, in mice overexpressing hFAM173B in sensory neurons, the reduction in weight bearing remained for at least 19 days after carrageenan (Fig 2F). Moreover, sensory FAM173B expression induced spontaneous pain, measured using a conditioned place preference (CPP) test [34, 35], that was present 1 month after intraplantar carrageenan injection (Fig 2G). Overall, these results indicate that FAM173B in sensory neurons promotes development of chronic pain. Next, we tested whether endogenous *mFam173b* mRNA expression levels are increased in the DRG during the persistent phase of CFA-induced inflammatory pain. At 1 week after intraplantar CFA injection, *mFam173b* mRNA expression in the DRG was increased compared to naive animals. In contrast, during acute inflammation at day 1 and 3 after CFA injections, *mFam173b* expression levels were indistinguishable from controls (S1N Fig), consistent with our previous findings [22].

### Characterization of FAM173B as a mitochondrial lysine-specific methyltransferase

**Fig 3:**
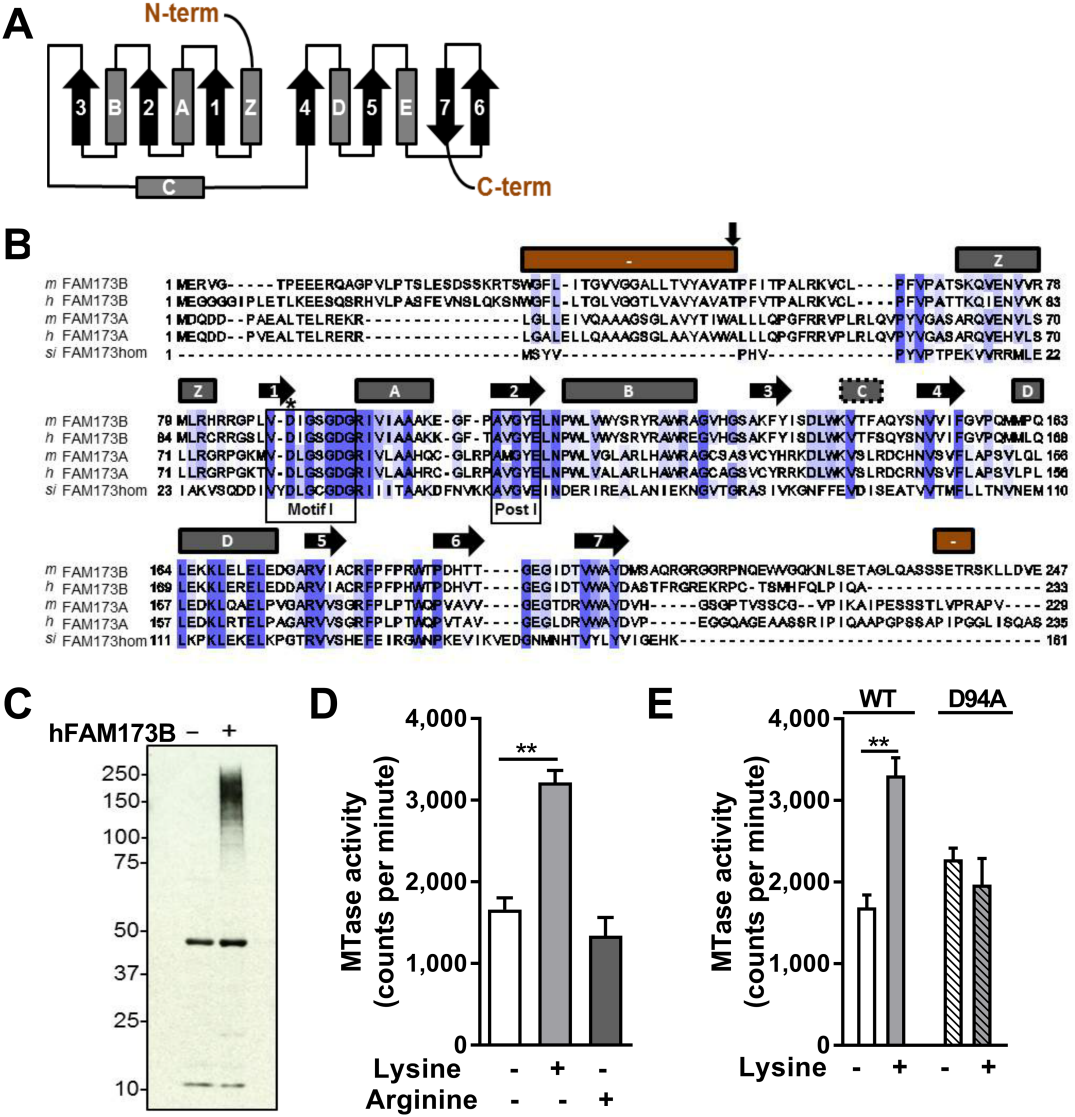
FAM173B is a lysine-specific methyltransferase. (A) Topology diagram of archetypical 7BS methyltransferase with alpha-helices (boxes) and β-strands (arrows). (B) Protein sequence alignment of FAM173A/B from *Homo sapiens* (h), *Mus musculus* (m), and the aKMT homolog of FAM173B (FAM173hom) from *Sulfolobus islandicus* (Si). Predicted secondary structure of mFam173b above alignment, coded as in A. Red bars indicate predicted N- and C-terminus of mFam173b. Motif I and Post I (boxed) are involved in binding of SAM. Asp94 (*) was mutated to generate an enzymatically inactive protein. The first residue (Thr56) in recombinant truncated hFAM173B (FAM173BΔ55) is also indicated (vertical arrow). (C) Fluorography of HEK293-extracts incubated with [^3^H]-SAM and recombinant hFAM173BΔ55. (D, E) WT FAM173BΔ55 (D, E) but not FAM173BΔ55-D94A (E) methylated lysine-homopolymers (*n* = 3 MTase reactions). Data are represented as mean ± SEM. * = *P* < 0.05; ** = *P* < 0.01. Statistical analyses were performed by one-way ANOVA followed by a post-hoc Holm-Sidak multiple comparison test (D) or by an unpaired two-tailed *t* test (E). Underlying data can be found in S1 Data. 7BS, 7 β-strands; HEK293, human embryonic kidney 293 cells; MTase, methyltransferase; SAM, *S*-adenosyl-L-methionine; WT, wild-type.

Bioinformatic analysis of FAM173B protein sequences show that FAM173B harbors characteristic motifs involved in binding of the methyl donor *S*-adenosyl-L-methionine (SAM). Moreover, it shows similarities for a subclass of methyltransferases characterized by a topology of 7 β-strands (7BS) (Fig 3A/3B) [36]. Human and mouse *FAM173B* are ubiquitously expressed (S2A/S2B Fig). An archaeal lysine-specific methyltransferase shows some homology with human FAM173B [37], therefore we explored whether hFAM173B specifically methylated lysine residues. To this end, we incubated a radioactive methyl donor, [^3^H]-SAM, with protein extracts of human embryonic kidney 293 cells (HEK293) together with purified recombinant hFAM173BΔ55 (without its putative transmembrane domain) and detected methyltransferase activity by fluorography. These experiments revealed hFAM173B-mediated methylation of high–molecular weight proteins (Fig 3C). To assess specificity of the enzyme, we evaluated homopolymers of lysine and arginine, the 2 most commonly methylated amino acid residues in proteins, as artificial substrates. When incubating recombinant hFAM173BΔ55 (Fig 3D) or full-length hFAM173B (S2C Fig) with [^3^H]-SAM and lysine or arginine homopolymers, hFAM173B displayed significant methyltransferase activity on poly-L-lysine but not on poly-L-arginine (Fig 3D). Importantly, a putatively enzymatically inactive mutant of hFAM173B (hFAM173B-D94A), generated by mutating a key conserved residue (Asp94) in the SAM-binding Motif I of hFAM173B (Fig 3B) [38], did not show significant methyltransferase activity (Fig 3E). The D94A mutation did not affect expression (S2D Fig).

**Fig 4:**
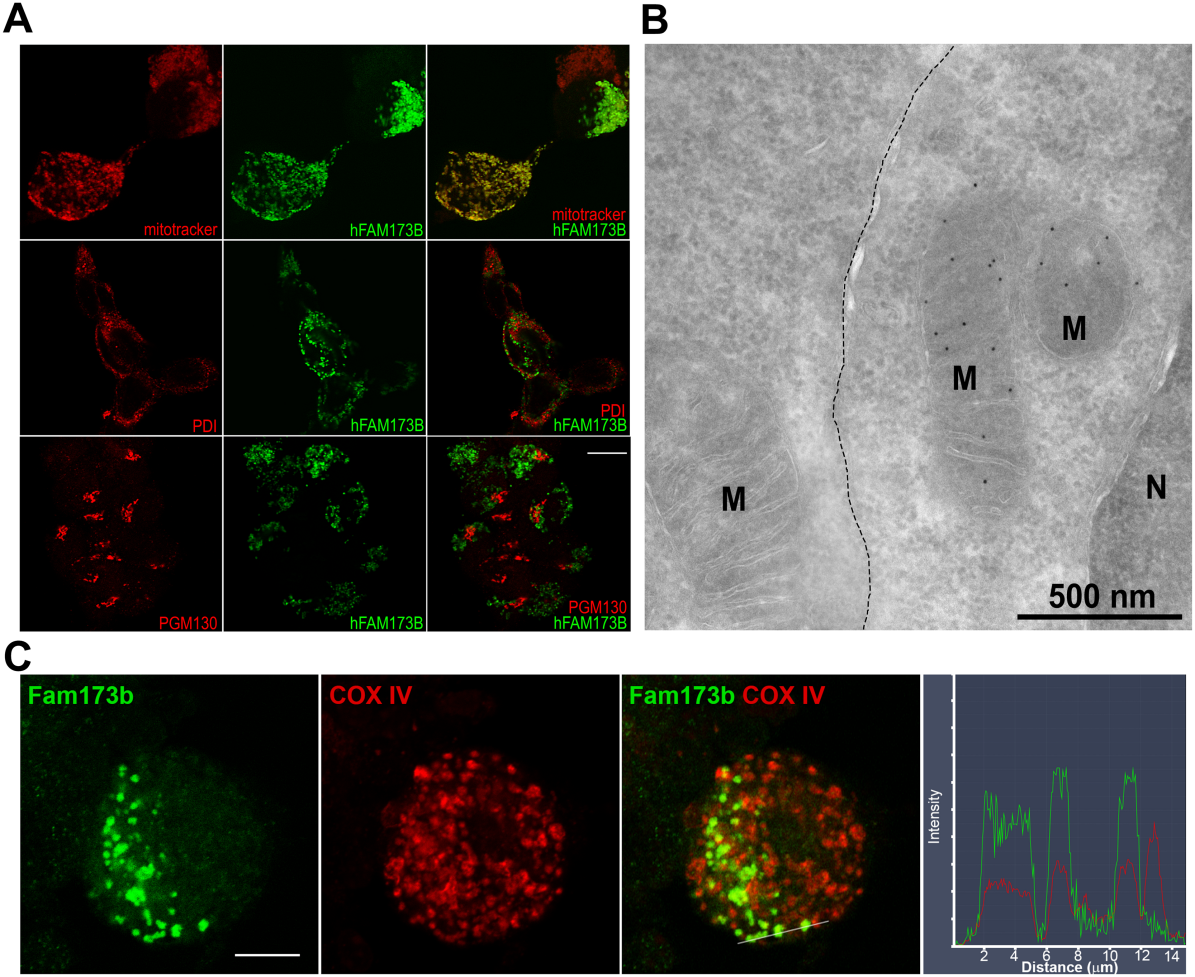
FAM173B is localized in mitochondria. (A) hFAM173B-GFP (green) colocalized with the mitochondrial dye MitoTrackerRedCMXROS but not with endoplasmic reticulum (PDI) or Golgi (PGM130) (all red). Scale bar 10 μm. (B) Electron microscopy of GFP-tagged, hFAM173B-expressing HEK293. Dotted line: boundary between nontransfected (left) and transfected cell (right). Scale bar 500 nm. (C) Cultured primary sensory neurons were stained for endogenous mFam173b and the mitochondrial marker COXIV. Right panel is the colocalization profile at the white line shown in panel 3 of the double immunostaining for mFam173b and COXIV. Scale bar 10 μm. COXIV, cytochrome c oxidase IV; HEK293, human embryonic kidney 293 cells; M, mitochondrion; N, nucleus; PDI, protein disulfide-isomerase.

To determine the subcellular localization of FAM173B, we expressed C-terminally GFP-tagged hFAM173B and mFam173b in Neuro2a (N2A), a neuronal cell line. Confocal imaging of GFP-tagged hFAM173B indicated that FAM173B colocalized with the mitochondrial dye MitoTrackerRedCMXROS but not with the endoplasmic reticulum marker protein disulfide-isomerase (PDI) or the Golgi scaffolding protein PGM130 (Fig 4A). The subcellular localization of mouse *Fam173b-GFP* and the methyltransferase-inactive mutant hFAM173B-D94A were also confined to mitochondria because they also colocalized with MitoTrackerRedCMXROS (S2E Fig). The localization of FAM173B and the methyltransferase death mutant FAM173B-D94A in mitochondria was further confirmed by western blot analysis of mitochondrial and cytosol fractions of N2A cells (S2F/S2G Fig). Electron microscopy of immunogold labeling of GFP-tagged hFAM173B showed that hFAM173B was predominantly present in the cristae of mitochondria when expressed in HEK293 (Fig 4B) or N2A cells (S2H Fig). Finally, endogenous mFam173b is located in mitochondria in cultured primary sensory neurons (Fig 4C).

### FAM173B and mitochondrial function

**Fig 5:**
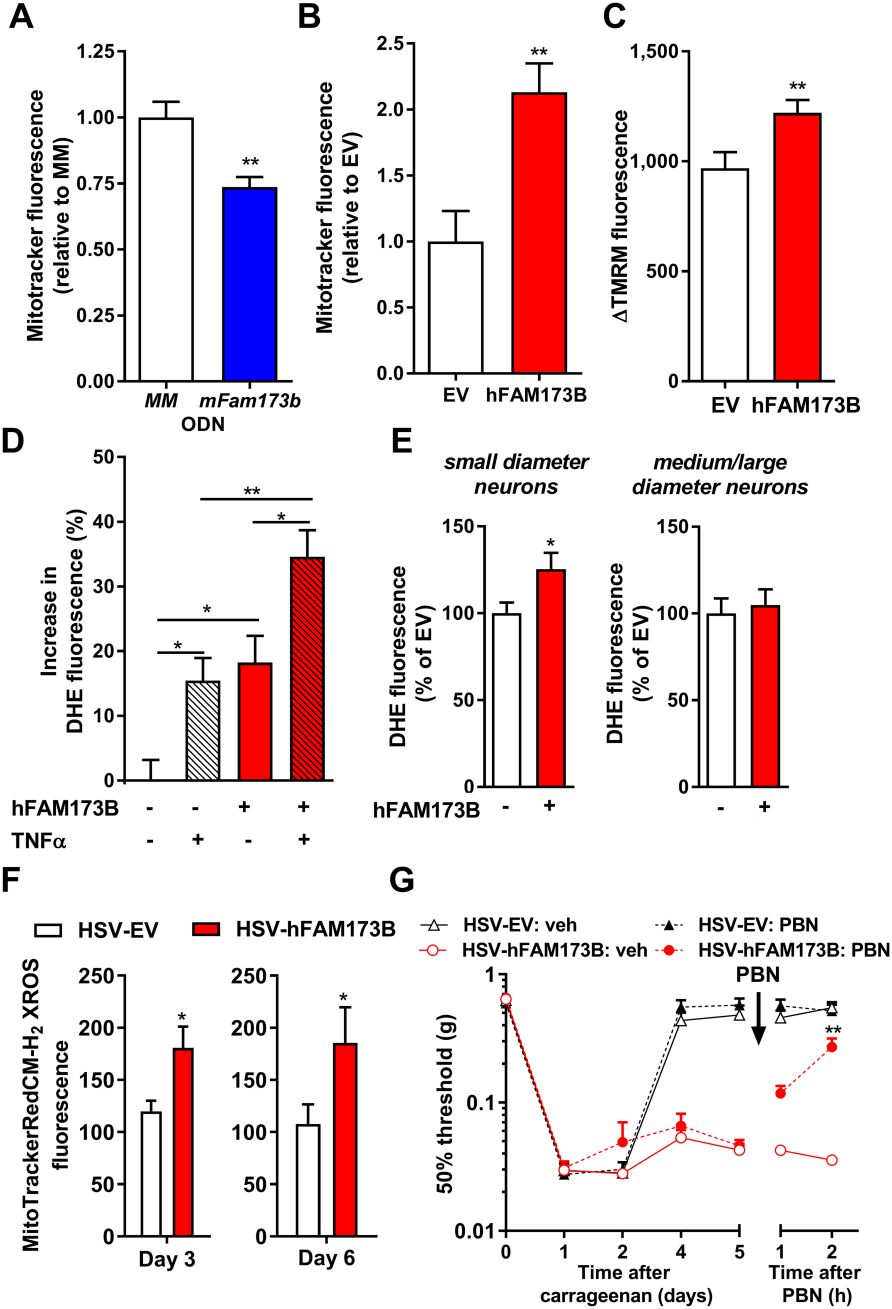
FAM173B and mitochondrial function. MitoTrackerRedCMXROS fluorescence, as a measure of mitochondrial potential, 48 hours after (A) mFam173b knockdown (MM-ODN *n* = 14; *mFAM173b-AS n* = 16 wells) or (B) hFAM173B overexpression (*n* = 7 wells) in N2A cells. (C) ΔTMRM fluorescence in HSV-mediated hFAM173B expression in cultured primary sensory neurons increased (*n* = 100–145 cells, 7 cultures). (D) HSV-mediated hFAM173B expression in cultured primary sensory neurons increased ROS production (DHE) after vehicle or 6 hours stimulation with 100 ng/ml TNFα (*n* = 90–130 cells, 9 cultures). (E–F) In vivo expression of hFAM173B in sensory neurons with HSV-hFAM173B prior to intraplantar carrageenan increased (E) DHE fluorescence intensity at day 5 (*n* = 7 mice) and (F) MitoTrackerRedCMH_2_-XROS fluorescence intensity at day 3 (*n* = 9 mice) and day 6 (EV *n* = 4, hFAM173B *n* = 6 mice) in small-diameter neurons. (G) Intraperitoneal injection of the ROS scavenger PBN attenuated the hFAM173B-mediated prolongation of carrageenan-induced mechanical hypersensitivity (*n* = 5 mice; HSV-FAM173B + PBN *n* = 6 mice). Data are represented as mean ± SEM. * = *P* < 0.05; ** = *P* < 0.01. Statistical analyses were performed by unpaired two-tailed t tests (A-C, E/F), by one-way (D) or two-way (G) repeated measures ANOVA followed by a post-hoc Holm-Sidak multiple comparison test. For exemplar pictures of A, B, and F, see S3 Fig. Blue bars indicate mFam173b knockdown and red bars/lines hFAM173B overexpression. Underlying data can be found in S1 Data. DHE, dihydroethidium; EV, empty vector; HSV, herpes simplex virus; MM-ODN, mismatch ODN; N2A, Neuro2a; ODN, oligodeoxynucleotide; PBN, phenyl-N-*t*-butylnitrone; ROS, reactive oxygen species; SEM, standard error of the mean; TNFα, tumor necrosis factor alpha.

To determine whether FAM173B modulates mitochondrial function, we assessed mitochondrial membrane potential (ΔΨm) [39]. Knockdown of mFam173b in N2A cells with *mFam173b-AS* (S3A Fig) reduced accumulation of the ΔΨm-sensitive dye MitoTrackerRedCMXROS compared to cells treated with control mismatch ODN (MM-ODN) (Fig 5A and S3B Fig), while overexpression of hFAM173B in N2A cells (S3D Fig) increased accumulation of MitoTrackerRedCMXROS (Fig 5B and S3C Fig). These data indicate that FAM173B promotes mitochondrial hyperpolarization. Similarly, overexpression of hFAM173B using HSV-hFAM173B amplicons in cultured primary sensory neurons (S3E Fig) or in N2A cells increased the difference in tetramethylrhodamine methyl ester (TMRM; a dye sequestered by active mitochondria in a ΔΨm-dependent manner [39]) fluorescence before and after the administration of the respiratory uncoupler carbonyl cyanide p-trifluoromethoxyphenylhydrazone (FCCP) (sensory neurons: Fig 5C; N2A: S3F Fig). This indicates that hFAM173B expression in sensory neurons hyperpolarizes mitochondria.

Mitochondrial hyperpolarization has been reported to cause increased ROS formation [40, 41]. Therefore, overexpression of hFAM173B may increase ROS formation in sensory neurons. Human FAM173B overexpression in N2A and HEK293 cells increased fluorescence of the ROS-sensitive dye dihydroethidium (DHE) [42], indicating that FAM173B promotes ROS formation in these cells (S3G/S3H Fig). Similarly, overexpression of hFAM173B in primary sensory neurons in vitro increased DHE fluorescence (Fig 5D). Stimulation of sensory neurons with the prototypic inflammatory mediator tumor necrosis factor α (TNFα) for 6 hours, known to promote ROS formation [43], enhanced DHE fluorescence, which was further increased when hFAM173B was expressed in sensory neurons with HSV-FAM173B (Fig 5D). Next, we addressed whether increased sensory neuron ROS formation also occurs during the hFAM173B-mediated switch from transient to persistent inflammatory pain in vivo. Expression of hFAM173B in sensory neurons increased DHE fluorescence in small (<25 μm)-diameter neurons that are central in inflammatory pain [44], but not in medium- and/or large-diameter neurons (>25 μm), 5 days after intraplantar carrageenan injection (Fig 5E). Next, we assessed whether FAM173B promotes mitochondrial superoxide production in vitro and in vivo. Overexpression of hFAM173B significantly increased fluorescence of the mitochondrial superoxide sensor MitoSox in N2A cells (S3I Fig). In vivo, HSV-mediated expression of hFAM173B in sensory neurons increased MitoTrackerRedCM-H_2_XROS fluorescence in small (<25 μm)-diameter neurons 3 and 6 days after intraplantar carrageenan injection (Fig 5F and S3J/S3K Fig), indicating that hFAM173B expression in sensory neurons promotes ongoing mitochondrial superoxide production in vivo. To assess whether the increased ROS production in sensory neurons contributes to hFAM173B-mediated prolongation of inflammatory pain, we administered the ROS scavenger phenyl-N-*t*-butylnitrone (PBN) during hFAM173B-induced persistent inflammatory hyperalgesia. PBN administration at day 5 after intraplantar carrageenan completely reversed the persistent carrageen-induced mechanical hyperalgesia (Fig 5G) in mice expressing hFAM173B in sensory neurons. PBN administration did not affect mechanical thresholds in mice treated with control HSV (Fig 5G). These data indicate that sensory neuron FAM173B-mediated prolongation of inflammation-induced hypersensitivity is maintained through an ROS-dependent pathway.

### FAM173B-induced microglia activation

**Fig 6:**
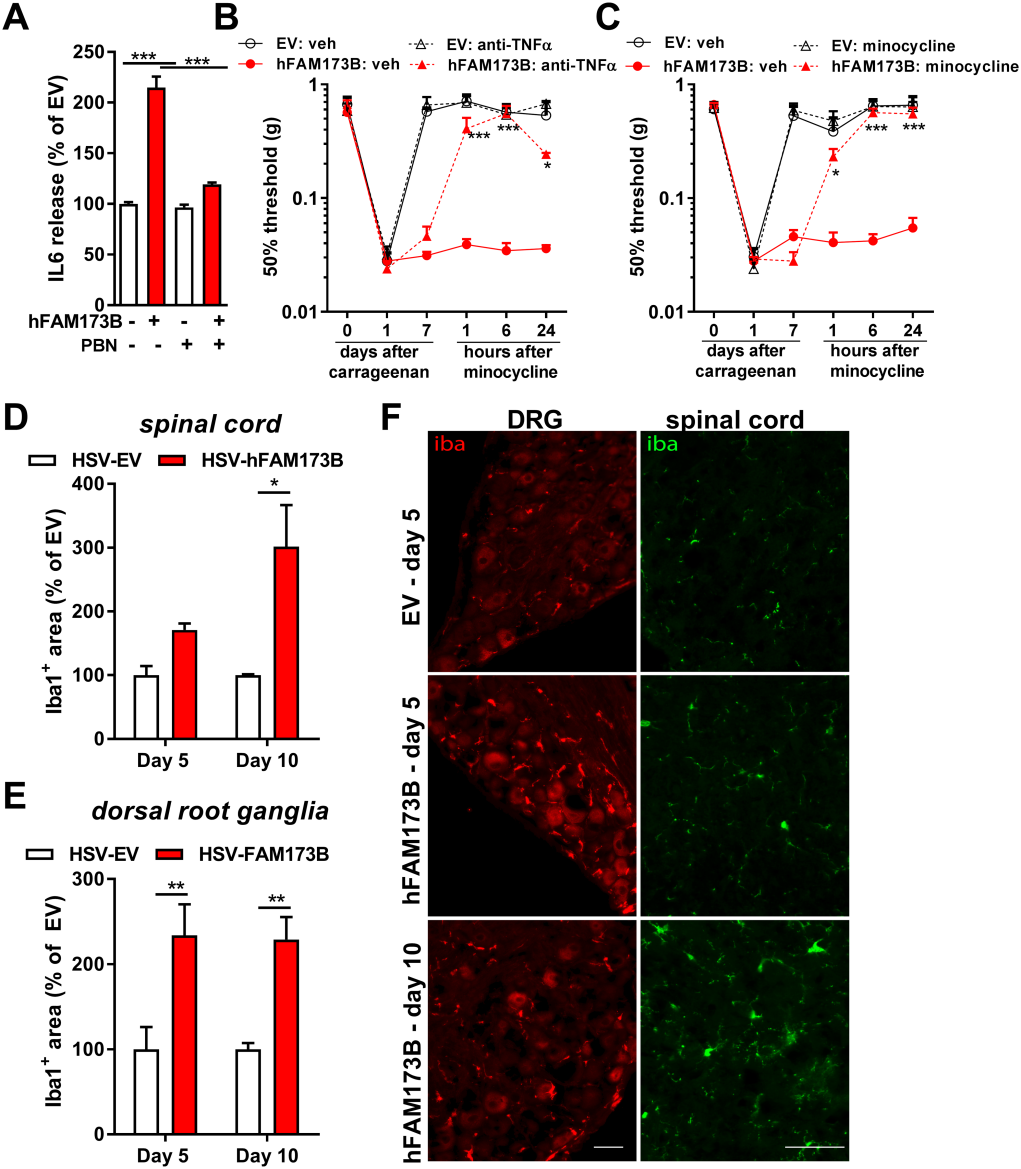
FAM173B-induced microglia activation. (A) IL6 release by spinal microglia after stimulation for 24 hours with supernatants of hFAM173B-expressing sensory neurons that were previously stimulated with 100 ng/ml TNFα +/- PBN (2 mM; ROS scavenger) for 6 hours, washed, and cultured for a subsequent 15 hours to capture sensory neuron–derived factors (EV-PBN *n* = 11; FAM173B-PBN *n* = 7; EV+PBN and FAM173B+PBN *n* = 3 wells; 100% = 59.4 pg/ml based on 3 independent experiments). (B) Intrathecal anti-TNFα (100 μg/mouse, HSV-FAM173B: *n* = 6; HSV-EV: *n* = 6 mice) or (C) minocycline (30 μg/mouse, minocycline: *n* = 12, vehicle: *n* = 6 mice) injection 7 days after intraplantar carrageenan attenuates hFAM173B-mediated prolongation of carrageenan-induced hyperalgesia. (D–E) Intraplantar HSV-hFAM173B injection prior to induction of paw inflammation increased Iba1-positive area in (D) spinal cord and (E) DRG at day 5 (*n* = 4 mice) and day 10 (*n* = 6 mice) after intraplantar carrageenan injection. (F) Exemplar images of quantified Iba1 staining in D and E. Scale bars 50 μm. Data are represented as mean ± SEM. * = *P* < 0.05; ** = *P* < 0.01; *** = *P* < 0.001. Statistical analyses were performed by one-way ANOVA (A) or by two-way repeated measures ANOVA (B–E) followed by a post-hoc Holm-Sidak multiple comparison test. Underlying data can be found in S1 Data. DRG, dorsal root ganglia; EV, empty vector; HSV, herpes simplex virus; Iba1, ionized calcium binding adaptor molecule 1; IL6, interleukin 6; PBN, phenyl-N-*t*-butylnitrone; ROS, reactive oxygen species; SEM, standard error of the mean; TNFα, tumor necrosis factor α.

Microglia/macrophage activation and the production of proinflammatory mediators in the spinal cord/DRG play a key role during persistent pain, including persistent inflammatory pain [7, 10, 12, 45, 46]. ROS formation can initiate proinflammatory cascades and activate microglia in the central nervous system [14]. As a next step, we evaluated whether FAM173B expression in primary sensory neurons promotes the ability of sensory neurons to activate glial cells in vitro in an ROS-dependent manner. Primary sensory neuron cultures were stimulated with 100 ng/ml TNFα [47] for 6 hours with or without the ROS scavenger PBN. After the 6 hours, cells were washed extensively to remove TNFα and then further cultured overnight for 15 hours to capture sensory neuron–derived factors that could drive glial cell activation. The supernatants of unstimulated sensory neurons with or without overexpressing hFAM173B did not trigger microglia to release detectable levels of interleukin 6 (IL6) and TNFα (Fig 6A). However, incubation of primary spinal microglia with the supernatant of these TNFα-stimulated sensory neurons for 24 h promoted microglia to release IL6, which was strongly enhanced by overexpression of hFAM173B in sensory neurons and completely abolished by incubating sensory neurons with the ROS scavenger PBN during TNFα stimulation (Fig 6A). Overexpression of hFAM173B in sensory neurons also increased TNFα release by microglia (S4A Fig). IL6 and TNFα were not detectable in the conditioned medium or in supernatants of unstimulated microglia, indicating that IL6 and TNFα were released by microglia and not already present in sensory neuron cultures. These in vitro data indicate that hFAM173B expression in TNFα-stimulated sensory neurons promotes the release of glial cell–activating factors in an ROS-dependent manner. To test whether in vivo sensory neuron FAM173B promotes the engagement of microglia and subsequent TNFα release to drive ongoing inflammatory pain, we inhibited TNFα signaling and microglia activity in the spinal cord and DRG by intrathecal injection of a neutralizing anti-TNFα antibody and glial cell inhibitor minocycline, respectively. Intrathecal injection of the neutralizing anti-TNFα antibody at day 7 after intraplantar carrageenan inhibited the sensory neuron–specific, hFAM173B-mediated persistent inflammatory pain (Fig 6B and S4B Fig). Intrathecal injection of minocycline at day 7 after intraplantar carrageenan completely inhibited hFAM173B-induced persistent inflammatory hyperalgesia (Fig 6C and S4C Fig). To further validate the contribution of microglia to FAM173B-mediated prolongation of inflammatory pain, we investigated whether in vivo overexpression of hFAM173B engages glial cells after induction of inflammatory pain. Expression of hFAM173B specifically in sensory neurons increased the Iba1-positive immunofluorescence in DRG and spinal cord at 5 and 10 days after carrageenan treatment compared to mice treated with empty HSV amplicons (Fig 6D–6F, S4D Fig). This neuronal, FAM173B-mediated spinal microglia activation in vivo was attenuated after scavenging ROS with PBN (S4E Fig).

**Fig 7:**
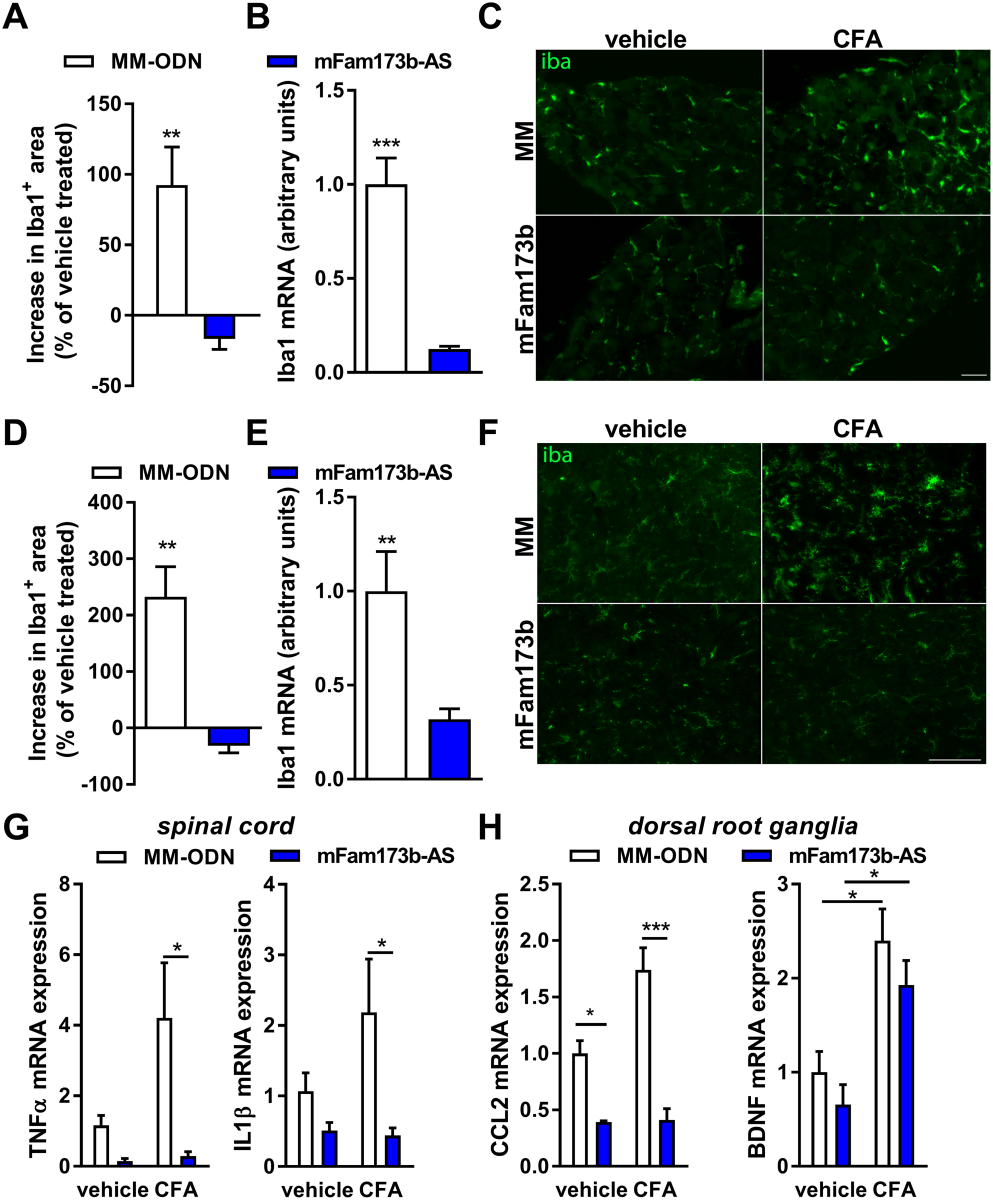
FAM173B knockdown prevents microglia activation. (A–H) Mice received intraplantar CFA to induce persistent hyperalgesia and received intrathecal *mFam173b-AS* to knockdown mFam173b or MM-ODN as control at day 5, 6, 7, 9, and 10. At day 11 after CFA injection, microglia activation in (A–C) DRG and (D–F) spinal cord was assessed by analysis of (A, D) fluorescent Iba1-positive area (*n* = 4 mice) and (B, E) Iba1 mRNA (*n* = 8 mice) in the dorsal horn of spinal cord or DRG. (C, F) Exemplar images of Iba1 staining of (C) DRG and (F) spinal cord as quantified in A and D. Scale bars 50 μm. The specific area quantified in the spinal cord is shown in S4D Fig. (G, H) Inflammatory mediator mRNA expression 24 hours after the last intrathecal injection of *mFam173b-AS* to knockdown mFam173b (day 11 after CFA) in (G) spinal cord and (H) DRG (*n* = 8 mice). Data are represented as mean ± SEM. * = *P* < 0.05; ** = *P* < 0.01; *** = *P* < 0.001. Statistical analyses were performed by unpaired two-tailed *t* tests ([A, B], [D, E]), by one-way ANOVA (G/H) followed by a post-hoc Holm-Sidak multiple comparison test. Underlying data can be found in S1 Data. CFA, complete Freund’s adjuvant; DRG, dorsal root ganglia; Iba1, ionized calcium binding adaptor molecule 1; MM-ODN, mismatch ODN; ODN, oligodeoxynucleotide; SEM, standard error of the mean.

Conversely, ODN-mediated knockdown of mFam173b prevented activation of glial cells during CFA-induced persistent pain as shown by the reversion of the CFA-induced increase in Iba1-positive area and mRNA expression in DRG (Fig 7A–7C) and spinal cord (Fig 7D–7F). *mFam173b-AS* treatment during CFA-induced persistent hyperalgesia did not affect the astroglial *GFAP* mRNA expression in the spinal cord and DRG (S4F/S4G Fig). The reduced signs of glia activation were associated with reduced expression of inflammatory mediators in the spinal cord and DRG known to play a role in persistent pain states [8]. Knockdown of mFam173b at day 5 to 11 after intraplantar CFA prevented the CFA-induced increase in *TNFα* and *IL1β* mRNA expression in the spinal cord (Fig 7G). In the DRG, mFam173b knockdown prevented the CFA-induced expression of the chemokine (C-C motif) ligand 2 (*CCL2*) but not of the growth factor Brain-derived neurotrophic factor (*BDNF*) (Fig 7H). Overall, these data indicate that neuronal FAM173B drives the persistence of inflammatory hyperalgesia through an ROS-dependent activation of glial cells.

### FAM173B methyltransferase activity and persistent pain

**Fig 8:**
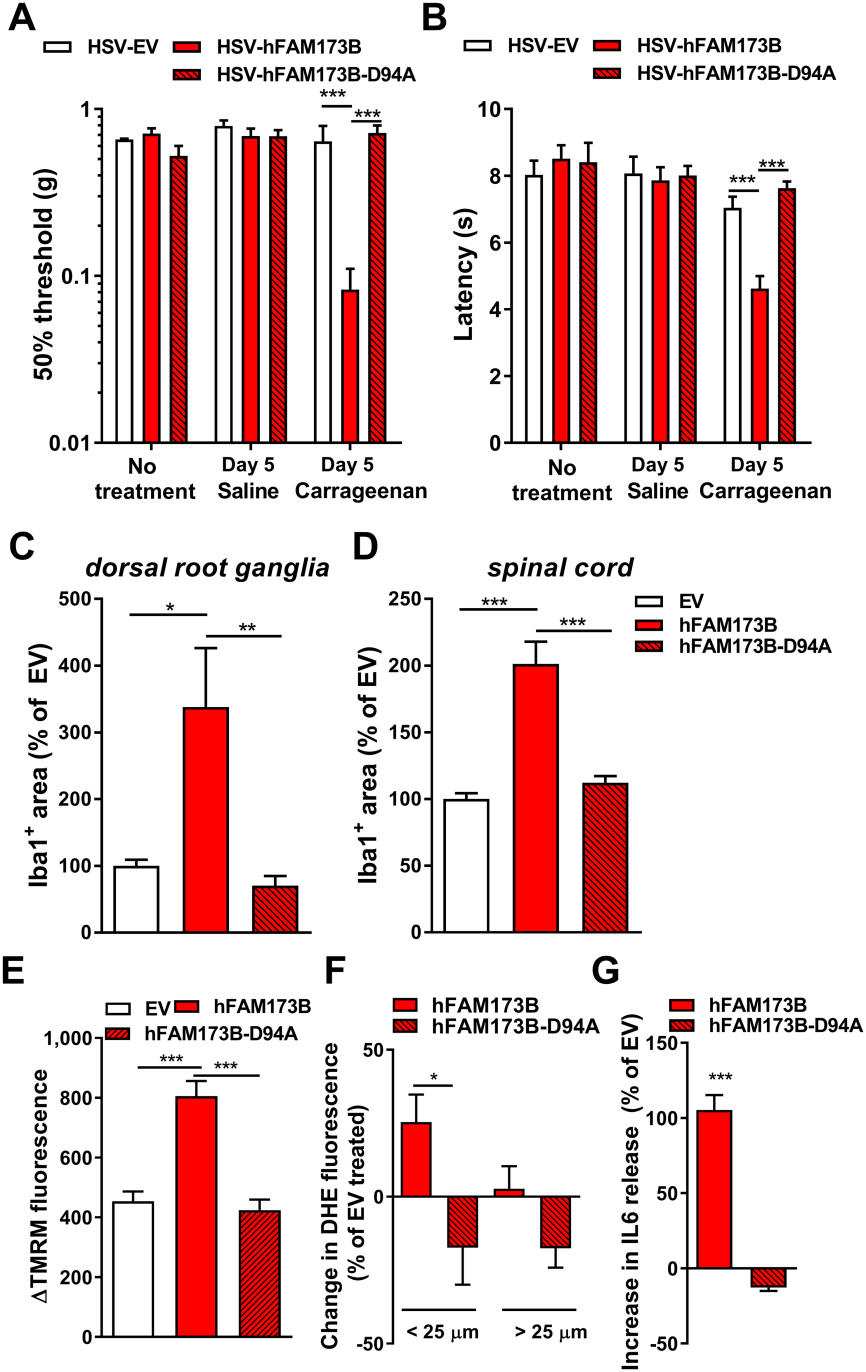
Methyltransferase activity requirement. WT hFAM173B but not hFAM173B-D94A expression in sensory neurons prolonged carrageenan-induced (A) mechanical and (B) thermal hypersensitivity (*n* = 6 mice). (C–F) Compared to WT hFAM173B, the methyltransferase-inactive mutant hFAM173B-D94A did not increase Iba1-positive area in (C) DRG and (D) dorsal horn of the spinal cord (*n* = 5 mice) at day 5 after intraplantar carrageenan injection, or enhance (E) ΔTMRM fluorescence in N2A in vitro (*n* = 115–150 cells) and (F) ROS production in small-diameter neurons in vivo (hFAM173B *n* = 7; hFAM173B-D94A *n* = 5 mice) at 5 days after intraplantar carrageenan. (G) Supernatants of 15 h–cultured sensory neurons expressing hFAM173B-D94A that were stimulated with 100 ng/ml TNFα for 6 hours and subsequently washed did not enhance IL6 release in spinal microglia in vitro (hFAM173B *n* = 4; hFAM173B-D94A *n* = 8 wells). Data are represented as mean ± SEM. * = *P* < 0.05; ** = *P* < 0.01; *** = *P* < 0.001. Statistical analyses were performed by unpaired two-tailed *t* tests (F, G), one-way ANOVA (C–E), or by a two-way ANOVA (A, B) followed by a post-hoc Holm-Sidak multiple comparison test. Underlying data can be found in S1 Data. DRG, dorsal root ganglia; Iba1, ionized calcium binding adaptor molecule 1; IL6, interleukin 6; N2A, Neuro2a; ROS, reactive oxygen species; SEM, standard error of the mean; TMRM, tetramethylrhodamine methyl ester; TNFα, tumor necrosis factor α; WT, wild-type.

**Fig 9:**
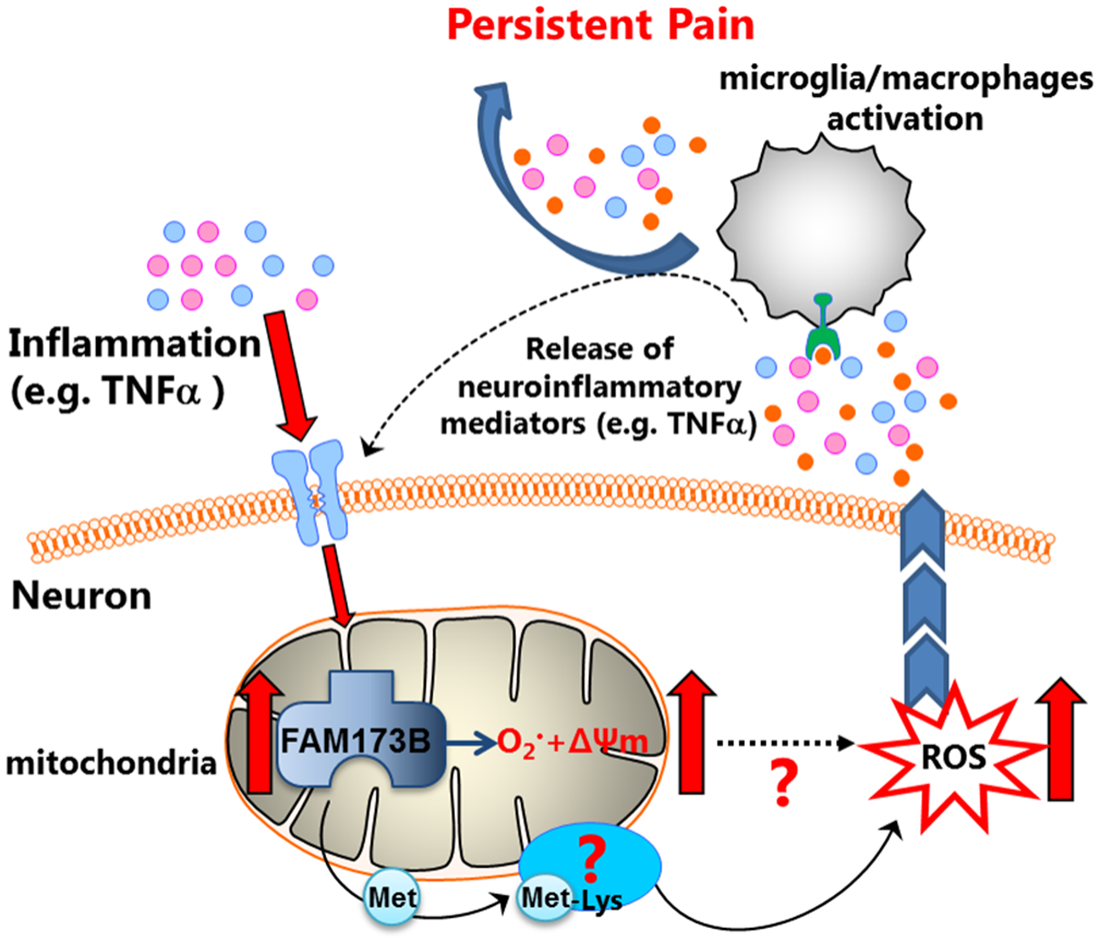
Proposed model of FAM173B. Inflammation increases FAM173B expression. Mitochondrial FAM173B methylates lysine(s) on a yet unknown substrate(s), causes mitochondrial hyperpolarization (ΔΨm↑), and increases superoxide and ROS production, which activates glial cells, thereby promoting persistent inflammatory pain. ΔΨm, mitochondrial membrane potential; ROS, reactive oxygen species.

We next determined whether the methyltransferase activity of FAM173B in sensory neurons is required to regulate chronic inflammatory pain through ROS- and glial cell–dependent mechanisms. Intraplantar (Fig 8A/8B) or intrathecal administration of HSV amplicons encoding for the methyltransferase-deficient mutant hFAM173B-D94A (S5A/S5B Fig) did not prolong carrageenan-induced thermal and mechanical hyperalgesia, while expression of wild-type (WT) hFAM173B prolonged transient inflammatory hyperalgesia (Fig 8A/8B and S5A/S5B Fig). In vivo overexpression of hFAM173B-D94A in sensory neurons prior to induction of inflammatory hyperalgesia did not increase Iba1-positive area in the DRG and spinal cord at day 5 after carrageenan injection (Fig 8C/8D and S5C Fig), indicating the requirement of FAM173B methyltransferase activity in sensory neurons to promote chronic pain and glial cell activity. In vitro, overexpression of hFAM173B-D94A did not affect ΔΨm, in contrast to WT hFAM173B, which increased ΔΨm (Figs 8E and 5A/5C). In addition, expression of WT hFAM173B but not hFAM173B-D94A increased the fluorescence of the ROS-sensitive dye DHE in small (<25 μm)-diameter neurons at day 5 during carrageenan-induced inflammatory hyperalgesia, indicating that FAM173B-mediated increase in ΔΨm and ROS production is also methyltransferase dependent (Fig 8F and S5D Fig). Finally, culturing spinal microglia with supernatants of TNFα-stimulated sensory neurons expressing WT FAM173B increased IL6 (Fig 8G) and TNFα (S5E Fig) release by microglia, while overexpressing hFAM173B-D94A had no such effect. Overall, these results indicate that the methyltransferase activity of FAM173B, and not the protein per se, is important to control the development of chronic pain through an ROS-dependent mechanism involving the activation of glial cells (Fig 9).

## Discussion

The precise mechanisms that lead to the development of persistent pain states remain to be fully uncovered. Here, we establish an important and completely novel role for FAM173B in sensory neurons in the development of chronic pain, identify its enzymatic function, and demonstrate a novel link between chronic pain and protein lysine methylation. First, insights came from a recent GWAS that identified a genomic region associated with chronic widespread pain and that included the *FAM173B* gene, which was functionally uncharacterized [22]. We show that *mFam173b* mRNA expression is increased in DRG during chronic inflammatory pain and knockdown of *mFam173b* expression abrogated persistent inflammatory and neuropathic pain. The localization of FAM173B in mitochondria and its effect on sensory neuron ΔΨm and ROS production highlights a unique lysine-methyltransferase–dependent pathway that regulates inflammation-induced hyperalgesia and spontaneous pain. In addition, we show that neuronal FAM173B methyltransferase activity promotes persistent ROS formation in sensory neurons after a transient peripheral inflammation leading to the engagement of microglia/macrophages in the spinal cord/DRG and persistent pain. Therefore, these data provide a mechanism by which FAM173B contributes to a novel pain pathway in chronic pain.

The 7BS class of methyltransferases represents a large group of enzymes that target a wide range of substrates, and several of these enzymes in humans have recently been established as lysine-specific protein methyltransferases [48, 49]. We show here that FAM173B harbors motifs characteristic of a 7BS methyltransferase and methylates lysine residues to promote chronic pain. A well-studied 7BS methyltransferase in relation with pain is catechol-O-methyltransferase (COMT), which inactivates biological active catechols; reduced COMT enzymatic activity contributes to reduced opioid analgesia and increased pain sensitivity [50, 51]. Other methyltransferases, such as DNA and histone methyltransferases, modify neuronal morphology, activity, and synaptic plasticity to induce pain hypersensitivity in chronic pain conditions via epigenetic modifications [52, 53]. FAM173B is different from known pain-promoting methyltransferases because it localizes to mitochondrial cristae and methylates lysine residues in high–molecular weight proteins. Therefore, FAM173B belongs to a unique class of 7BS mitochondrial lysine-specific methyltransferase and promotes ROS production in neurons leading to persistent pain.

Mitochondria are essential for adenosine triphosphate (ATP) generation, calcium buffering, and ROS generation in sensory neurons [13]. Mitochondrial dysfunction plays a role in many neurological disorders such as Parkinson disease, Alzheimer disease, and Huntington disease [54–56], but the role of mitochondria in pain is relatively little explored [13]. Mitochondrial dysfunction contributes to painful peripheral neuropathies evoked by diabetes, chemotherapy, and trauma-induced nerve injury in humans and rodents [13, 15, 57, 58]. Recent studies also highlight a link between sensory neuron mitochondrial abnormalities and chronic inflammatory pain development. A data-independent acquisition mass spectrometry of the DRG proteome during CFA-induced inflammatory pain showed differential expression of a multitude of proteins involved in mitochondrial functioning. Inhibition of mitochondrial functioning in vivo during CFA-induced inflammatory pain using rotenone, a mitochondrial complex I inhibitor, diminished the inflammation-induced hyperalgesia [59]. Moreover, neuropathic and inflammatory pain is associated with increased mitochondrial oxygen consumption in the sciatic nerve and increased superoxide production in the spinal cord, respectively [57, 60]. Interestingly, signs of mitochondrial dysfunction and ROS production are also observed in patients with complex regional pain syndrome and fibromyalgia, including individuals with chronic widespread pain [19, 20]. Although ROS are thought to be central in chronic pain conditions, clinical trials with antioxidant therapies have been disappointing [61, 62]. The exact reasons why they fail are not known, but antioxidant treatments likely do not scavenge ROS directly at the intracellular source, preventing full inhibition of ROS-dependent pathways. Therefore, there is a need to identify critical upstream processes of ROS production in chronic pain that may represent better targets to inhibit these ROS-dependent processes leading to pain. Here, we identified FAM173B as a methyltransferase that, when overexpressed, hyperpolarizes mitochondria and promotes mitochondrial and neuronal (cytosolic) ROS production after peripheral inflammation, leading to the engagement of microglia and persistence of inflammatory pain. The question remains whether the observed increase in cytosolic ROS formation after hFAM173B overexpression is a consequence of the FAM173B-induced mitochondrial superoxide production or whether it is caused by other cytosolic sources such as oxidants producing peroxisomes or endoplasmic reticula [63, 64]. FAM173B may represent an important upstream factor of persistent ROS production in sensory neurons leading to pain. As such, FAM173B could represent the long-sought therapeutic target upstream of ROS production to treat persistent pain. However, FAM173B is ubiquitously expressed, thus potentially dampening its potential as therapeutic target. Nevertheless, FAM173B expression increases in sensory neurons through yet unknown mechanisms during inflammatory pain. Moreover, a recent, large, whole-genome sequencing study of an Icelandic population demonstrated that individuals deficient for FAM173B were healthy, suggesting that targeting FAM173B may be feasible [65]. The requirement of methyltransferase activity of FAM173B in chronic pain development demonstrates that inhibiting FAM173B activity is a potential strategy to inhibit chronic pain. It will be important to identify the substrate(s) that is/are methylated by FAM173B to modulate mitochondrial functioning, including mitochondrial respiration, ΔΨm, and mitochondrial superoxide production in sensory neurons.

The GWAS pointed to a role of FAM173B in chronic widespread pain, and our findings show that FAM173B plays a critical role in inflammatory and neuropathic pain. Currently, no animal models exist for chronic widespread pain. However, chronic widespread pain may have features of both inflammatory and neuropathic pain [66–70]. Therefore, our data indicating that FAM173B is involved in inflammatory and neuropathic pain pathways are likely to also have relevance for chronic widespread pain. Further studies are required to develop animal models of chronic widespread pain and test for the role of FAM173B activity in these models before clinical translation to chronic widespread pain patients should be considered.

We show here that sensory neuron FAM173B methyltransferase activity causes the engagement of spinal microglia in a model of transient inflammatory pain. The contribution of microglia to persistent pain states is well established [7, 10], and several neuron-derived signals contributing to spinal cord microglia activation in persistent pain models have been identified, including fractalkine, ATP, monocyte chemoattractant protein 1 (MCP1), colony-stimulating factor 1, and several neurotransmitters [6, 11, 12]. However, the molecular determinants in sensory neurons that trigger these cells to release substances to engage microglia are not well known. Here, we show that FAM173B methyltransferase activity in sensory neurons determines whether spinal cord microglia are engaged during peripheral inflammatory conditions in vivo. In vitro, the expression of FAM173B in sensory neurons promoted the release of glial cell–activating factors in an ROS-dependent manner after stimulation of sensory neurons with the proinflammatory cytokine TNFα.

In conclusion, we propose that the mode of action by which FAM173B promotes chronic pain is through its lysine-specific methyltransferase activity in mitochondria, promoting ROS production in sensory neurons, resulting in glial cell engagement. These data provide a conceptual framework to explain a potential role of FAM173B as a chronic pain protein in humans and open the possibility for inhibitors of FAM173B methyltransferase activity to treat chronic pain.

## Materials and methods

### Ethics statement

All experiments were performed in accordance with international guidelines and approved by the experimental animal committee of University Medical Center Utrecht (2012.I.05.068, 2014.I.06.042) or approved by the national Central Authority for Scientific Procedures on Animals (CCD) and the local experimental animal welfare body (AVD115002015323).

### Animals

Mice were maintained in the animal facility of the University of Utrecht. Experiments were conducted using both male and female (aged 8–12 weeks) C57BL/6 mice (Harlan Laboratories, Indianapolis, IN, US) because we did observe not overt sex differences during pain behavior measurements. Mice were housed in groups under a 12:12 light dark cycle, with food and water available ad libitum. The home cages contained environmental enrichments, including tissue papers and shelter. Mice were acclimatized for at least 1 week prior to the start of experiment. Sample sizes were calculated with power analysis at the time of the design of experiments. Mice received an intraplantar injection unilateral or in both hind paws of 5 μl λ-carrageenan (1% w/v, Sigma-Aldrich, St. Louis, MO, US) to induce transient inflammatory pain [27] or 20 μl CFA (Sigma-Aldrich, St. Louis, MO, US) to induce persistent inflammatory pain [29]. SNI was performed as described previously [30, 71]. Heat withdrawal latency times were determined using the Hargreaves test (IITC Life Science, Woodland Hills, CA, US) [72, 73]. Mechanical thresholds were determined using the von Frey test (Stoelting, Wood Dale, IL, US) with the up-and-down method as we described [72, 74]. Changes in weight bearing were evaluated using a dynamic weight bearing (DWB) apparatus (Bioseb, Vitrolles, France) as described [75]. The weight bearing of the affected paw was calculated as ratio of the weight between the affected paw and total weight and expressed relative to baseline. To assess persistent nonevoked pain behavior, we used the CPP test as described previously [34, 35]. In short, CPP (Stoelting, Wood Dale, IL, US) was calculated by subtracting the mean time spent in the white room during preconditioning (days 1 and 2) from the time spent in the white room (day 5) after 2 days of conditioning (day 3–4) with intraperitoneal injections of gabapentin (100 mg/kg, Sigma-Aldrich, St. Louis, MO, US) as has been described before. CPP was applied 1 month after induction of a transient inflammation, and hyperalgesia was followed prior to CPP using Hargreaves and Von Frey tests. In experiments in which mice received intraplantar injections, latency times and 50% thresholds of left and right paws were considered as an independent measure, while in experiments with intrathecal or intraperitoneal drug administration, the average of the left and right paw were considered as an independent measure. To minimize bias, animals were randomly assigned to the different groups prior to the start of experiment, and all experiments were performed by experimenters blinded to treatment. After pain behavior assessments, mice were brought back to their home cages to minimize discomfort. At the end of the experiments, mice were euthanized by cervical dislocation.

### DNA and viral constructs

Full-length *mFam173b* (NM_ 026546.1) and *hFAM173B* (NM_199133.3) were cloned into several vectors, including pAcGFP-N1, pIRES2-AcGFP1, bacterial expression vector pET28a, and pCMV6 containing a myc-tag at the C-terminal of human or mouse *FAM173B* (Origene, Rockville, MD, US). pIRES2-AcGFP vectors were used for functional experiments, and GFP expression was used to verify successful transfection. The pCMV6 and pAcGFP-N1 vectors were used for identification of cellular and subcellular localization of FAM173B, and pET28a was used for the production of recombinant FAM173B in *Escherichia coli*.

We generated a bicistronic HSV construct by cloning *hFAM173B* or *hFAM173B-D94A* in which residue (Asp94) is mutated to alanine in order to generate enzymatically inactive protein [38], under control of the α4 promotor and with GFP under control of the α22 promoter [27]. Control HSV-EV only expresses GFP. HSV was produced as previously described [76]. Mice were inoculated twice (day −3 and day −1 prior to carrageenan or at day 5 and 7 after CFA) with 2.5 μl of 1.4 × 10^7^ pfu/ml (intraplantar) or 5 μl 5 × 10^6^ pfu/ml (intrathecal).

### Drug administration

For behavioral analysis, mice received an intraperitoneal injection (day 5 after carrageenan) with 100 μl PBN (100 mg/kg, Sigma-Aldrich, St. Louis, MO, US). For spinal cord analysis, mice received 2 PBN injections (2 hours apart) at 1 month after carrageenan. Spinal cords were collected 2 hours after the last PBN administration. Intrathecal injections (5 μl) with minocycline (6 μg/μl, Sigma-Aldrich, St. Louis, MO, US), neutralizing TNFα antibody (20 μg/μl, Enbrel), and (Cy3-labeled, set1) ODN (3 μg/μl day 5, 6, 7, 9, and 10, Sigma Aldrich, St. Louis, MO, US) were performed under light isoflurane anesthesia as described [72, 77]. The following phosphorothioated ODN sequences that specifically target *mFam173b* and not *hFAM173B* were used:

$$\begin{array}{ll} \text{Set}\;1\;Fam173b:\text{CCCgCCTgTCTTTCTTCCTC} & \text{MM}:\text{CgCCTCCgTTCCTTTCTCCT}\\ \text{Set}\;2\;Fam173b:\text{gggTCCTCTTCTgTgTCgCA} & \text{MM}:\text{gTgCTCgTCTTgCCgACgCT} \end{array}$$

### Cell lines, primary cell cultures, and transfections

HEK293 and mouse neuroblastoma N2A cells were kept in Dulbecco’s Modified Eagle medium (DMEM) with Glutamax-l containing 4.5 g/L D-Glucose, pyruvate, and 10% fetal calf serum. FAM173B expression was down-regulated (100 μM ODN) or overexpressed with plasmids as described above using Lipofectamin 2000 (Life Technologies, Waltham, MA, US) according to manufacturer’s instructions. For measuring ΔΨm, the cells were incubated for 20 to 30 minutes with 50 nM MitoTrackerRedCMXROS (Life Technologies, Waltham, MA, US) or 50 nM TMRM (Sigma-Aldrich, St. Louis, MO, US) 2 days after transfection and following manufacturer’s instructions. Cells were fixed with 4% paraformaldehyde (PFA) after MitoTrackerRedCMXROS or directly imaged without fixation (TMRM experiments). For the TMRM experiments, fluorescence was captured before and after the addition of the respiratory uncoupler FCCP that abolishes ΔΨm without affecting cell membrane potential [39]. The ΔTMRM fluorescence was calculated by measuring difference in the TMRM fluorescence before and after FCCP administration. Fluorescence was captured using AxioCAM MRm from Zeiss Axio Observer microscope and analyzed with ImageJ software.

DRG were collected, and primary sensory neurons were cultured as described [25]. Twenty-four hours after plating, sensory neuron cultures were inoculated with HSV (10,000 pfu) for 3 days. The antimitotic fluoro-deoxyuridine (FDU; 13.3 μg/ml, Sigma-Aldrich, St. Louis, MO, US) was added to inhibit satellite glial cell growth in the neuronal cultures. Sensory neurons were stimulated with 100 ng recombinant TNFα (Peprotech, Rocky Hill, NJ, US) with or without PBN (2 mM, Sigma-Aldrich, St. Louis, MO, US). Six hours after neuronal TNFα stimulation (+/− PBN), the cultures were washed 3 times with media (DMEM), and new media was added; after 15 hours, supernatants were collected. The collected supernatants were diluted 1:1 with DMEM and added to spinal microglia cultures for 24 hours. Spinal microglia were cultured as described previously [78]. After collection of the supernatants, IL6 and TNFα contents were determined by ELISA according to manufacturer’s protocol (R&D Systems, Minneapolis, MN, US). The detection limit of IL6 was 15 pg/ml and of TNFα 31 pg/ml.

### Electron microscopy

HEK293 and N2A cells were grown in 6-well plates and transfected with pCMV6-FAM173Bmyc as described above. The cells were treated as described previously [79]. Briefly, cells were chemically fixed using 2% formaldehyde (FA), 0.2% glutaraldehyde in 0.1 M phosphate buffer pH 7.4 (Pi) for 2 hours, and stored overnight in 1% FA in Pi. After rinsing with PBS (3 times) and PBS 0.15 M glycine, a 1% gelatin solution was put on the cells, and the cells were removed from the Petri dish using a cell scraper, transferred to an Eppendorf vial, and spun down. The 1% gelatin was removed, and the cells were suspended in 12% gelatin at 37°C. After 10 min, the cells were spun down and the gelatin was allowed to solidify at 0°C. Small (0.5 × 0.5 × 0.5 mm) blocks were prepared and transferred to 2.3 M sucrose. After overnight infiltration of sucrose in a rotator, the blocks were mounted on specimen holders and frozen in liquid nitrogen. Ultrathin sections (70 nm) were prepared on a Leica UC7/FC7 (Leica, Vienna, Austria) at −120°C. Immunolabeling was performed with Rabbit anti-GFP (Acris Antibodies, Herford, Germany) and protein A-Gold (CMC, Utrecht, the Netherlands). The immunogold labeled sections were examined with a Tecnai 12 or 20 (FEI, Eindhoven, the Netherlands).

### ROS and superoxide detection

In vivo, DHE—to measure ROS formation (50 μM, 5 μl, Life technology, Waltham, USA)—or MitoTrackerRedCM-H_2_XROS, which fluoresces upon oxidation—to measure mitochondrial superoxide production (10 μl of 100 μM, Life Technologies, Waltham, MA) [80]—was injected intrathecal respectively at day 4 or day three/six after intraplantar carrageenan administration. Twenty-four hours later, mice were perfused with PBS and 4% PFA as described below, and DRGs were collected [18]. DHE and MitoTrackerRedCM-H_2_XROS fluorescence were analyzed in small-diameter neurons <25 μm and medium- and/or large-diameter neurons >25 μm.

For ROS or mitochondrial superoxide production measurements in vitro, primary sensory neurons or N2A were incubated with 10 μM DHE or 5 μM MitoSoX (Life Technologies, Waltham, MA) in HBSS for 20 minutes. After HBSS washes, cells were fixed with 4% PFA after DHE incubations or directly imaged without fixation (MitoSox experiments). Fluorescence was captured using AxioCAM MRm from Zeiss Axio Observer microscope and analyzed with ImageJ software.

### Immunostaining

Mice were deeply anesthetized with an overdose of sodium pentobarbital and transcardially perfused with PBS followed by 4% PFA, and spinal cords and DRGs were collected. Tissues were postfixed, cryoprotected in sucrose, embedded in OCT compound (Sakura, Zoeterwoude, the Netherlands), and frozen at −80°C. Cryosections (10 μm) of lumbar DRG and lumbar spinal cord segments L3–L5 were stained with anti-Iba1 (1:500, Wako Chemicals, Wako, Japan). DRGs were stained with anti-NF200 (1:200, Millipore, Bellerica, MA, US), biotinylated anti-IB4 (1:25, Vector Laboratories, Burlingame, CA, US), anti-F4/80 (1:500, Cedarlane, Burlington), and anti-GFAP (1:2000, Dako, Santa Clara, CA, US). N2A cells were stained with anti-PD1 (1:100, Enzo Life Sciences, Farmingdale, NY) and anti-pGM130 (1:100, BD Transduction Laboratories, San Jose, CA). Primary sensory neurons were stained with anti-FAM173B (1:500, biorbyt) and anti-COXIV (1:100, ThermoFisher Scientific, Waltham, MA). For the DRG, sciatic nerves, and hind paw stainings for FAM173B (1:500, Biorbyt, Cambridge, UK), GFP (1:3000, Abcam, Cambridge, UK), and peripherin (1:100, Sigma Aldrich, St. Louis, MO), tissues were fresh frozen, cut, and post-fixed in PFA prior for staining. Stainings were visualized by using alexafluor 488-(streptavidin) or 594-conjugated secondary antibodies. Nuclei were stained with 4′,6-diamidino-2-phenylindole (DAPI). Photographs were captured with a confocal laser scanning microscope LSM700 (colocalization experiments) or with a Zeiss Axio Observer microscope (Zeiss, Oberkochen, Germany) using identical exposure times for all slides within one experiment. Fluorescence intensity was analyzed with ImageJ software.

### Bioinformatics

*Homo sapiens* FAM173A (NP_076422.1) and FAM173B (NP_954584.2), *Mus musculus* Fam173a (NP_663385.2) and Fam173b (NP_080822.1), and the homolog of FAM173 proteins (FAM173hom) from the archaeal *Sulfolobus islandicus* (gi|227827841) were used for the alignment. The alignment was generated using the MUSCLE algorithm embedded in Jalview [81, 82], and prediction of protein secondary structure was performed with Jpred 3 [83].

### Expression and purification of recombinant FAM173B

Human full-length FAM173B, WT FAM173BΔ55 (without the putative transmembrane domain to avoid the formation of inclusion bodies), and FAM173BΔ55-D94A (enzymatically inactive) were cloned into pET28a and expressed as N-terminally hexahistidine tagged proteins in *E*. *coli* BL21-CodonPlus(DE3)-RIPL cells (Agilent, Santa Clara, CA) and purified using nickel-nitrilotriacetic acid-agarose (Qiagen, Hilden, Germany) according to manufacturer’s instructions and as described [38]. Eluted proteins were buffer exchanged [38], and protein purity was assessed by SDS-PAGE and Coomassie blue staining. Protein concentrations were measured using the Pierce BCA protein assay kit (Thermo Fisher Scientific, Waltham, MA).

### Methyltransferase assay

Methyltransferase reactions contained 10 μg of homopolymers or equivalent amounts of cell extracts from adenosine dialdehyde (AdOx)-treated HEK293 cells [84], [^3^H]-SAM (2 μCi), and recombinant hFAM173B (100 pmol) in 50-μl reactions and were incubated for 1 hour at 37°C, as described [49, 85]. Radioactivity in 10% trichloroacetic acid precipitated material was measured by scintillation counting, or proteins were resolved by SDS-PAGE and subjected to fluorography [49].

### Western blot

Isolation of mitochondria from N2A cells was performed with the Mitochondria Isolation Kit for Cultured Cells (ThermoFisher Scientific, Waltham, MA) according to manufacturer’s protocol. Protein concentrations of the total cell lysates or mitochondrial/cytosol fractions were determined using a Bradford assay (Bio-Rad, Hercules, CA). Protein samples (20 μg) were separated by 12% SDS-PAGE and transferred to a PVDF membrane (Immobilon-P, Millipore, Bellerica, MA). Membrane was stained with 1:1000 goat anti-FAM173B, 1:1000 mouse anti-COXIV (Invitrogen, Paisley, UK), or 1:1000 goat anti–β-actin, followed by incubation with 1:5000 donkey anti goat-HRP (others all Santa Cruz Biotechnology, Santa Cruz, CA). Specific bands were visualized by chemiluminescence (ECL, Advansta, Menlo Park, CA) and imaging system Proxima (Isogen Life Sciences, De Meern, the Netherlands).

### Real-time RT-PCR

Total RNA from freshly isolated DRGs and spinal cords was isolated using TRizol and RNeasy mini kit (Qiagen, Hilden, Germany). cDNA was synthesized using Reverse Transcriptase (Bio-Rad, Hercules, CA). Quantitative real-time PCR reaction was performed with an I-cycler iQ5 (Bio-Rad, Hercules, CA) as described [22]. We used the following primers:

$$\begin{array}{ll} mFam173b\;\text{forward}:\text{TggTgTgCCCCAgATgAT} & \text{reverse}:\text{TgCCCTCTCCAgTggTgT}\\ TNF\alpha \;\text{forward}:\text{gCggTgCCTATgTCTCAg} & \text{reverse}:\text{gCCATTTgggAACTTCTCATC}\\ IL1\beta \;\text{forward}:\text{CAACCAACAAgTgATATTCT} & \text{reverse}:\text{gATCCACACTCTCCAgCTgCA}\\ GFAP\;\text{forward}:\text{ACAgACTTTCTCCAACCTCCAg} & \text{reverse}:\text{CCTTCTgACACggATTTggT}\\ Iba1\;\text{forward}:\text{ggATTTgCAgggAggAAAAg} & \text{reverse}:\text{TgggATCATCgAggAATTg}\\ BDNF\;\text{forward}:\text{CACATTACCTTCCAgCATCT} & \text{reverse}:\text{ACCATAgTAAggAAAAggATgg}\\ CCL2\;\text{forward}:\text{ggTCCCTgTCATgCTTCTg} & \text{reverse}:\text{CATCTTgCTggTgAATgAgTAg}\\ GAPDH\;\text{forward}:\text{TgAAgCAggCATCTgAggg} & \text{reverse}:\text{CgAAggTggAAgAgTgggAg},\\ HPRT\;\text{forward}:\text{TCCTCCTCAgACCgCTTTT} & \text{reverse}:\text{CCTggTTCATCATCgCTAATC} \end{array}$$

Data were normalized for *GAPDH* and *HPRT* expression.

### Conventional PCR

cDNA was synthesized from 1 μg total RNA (Clontech, Mountain View, CA), and PCR was performed using Phusion polymerase (ThermoFisher Scientific, Waltham, MA) following manufacturing instructions. Human and mouse FAM173B were detected in a tissue panel (Clontech, Mountain View, CA) using the following primers:

$$\begin{array}{ll} hFAM173B\;\text{forward}:\text{gTAgCCACgCCgTTTgTAAC} & \text{reverse}:\text{CATCATCTgAggCACACCgA}\\ \beta -actin\;\text{forward}:\text{CCTggCACCCAgCACAAT} & \text{reverse}:\text{GggCCggACTCgTCATACT}\\ mFam173b\;\text{forward}:\text{TggTgTgCCCCAgATgAT} & \text{reverse}:\text{TgCCCTCTCCAgTggTgT}\\ HPRT\;\text{forward}:\text{TCCTCCTCAgACCgCTTTT} & \text{reverse}:\text{CCTggTTCATCATCgCTAATC} \end{array}$$

### Statistical analysis

All data are presented as mean ± SEM and were analyzed with GraphPad Prism version 7.02 using unpaired two-tailed t tests, one-way or two-way ANOVA, or as appropriate two-way repeated measures ANOVA, followed by post-hoc Holm-Sidak multiple comparison tests. A *P* value less than 0.05 was considered statistically significant, and each significance is indicated with * for *P* < 0.05, ** for *P* < 0.01, and *** for *P* < 0.001.

## Supporting information

S1 Data Spreadsheet. (XLSX)

S1 Fig FAM173B in sensory neurons promotes chronic pain. (A) Left images represent DRG of mice injected with either labeled (Cy3, red) or unlabeled mouse Fam173b antisense ODNs (*mFam173b-AS)*. Labeling is visible in sensory neurons as well as some cells surrounding the sensory neurons. Scale bar 50 μm. Right images: After intrathecal Cy3-labeled *mFam173b-AS* injections (red), lumbar DRG from mice were stained for IB4, NF200, Iba1, and GFAP (green), and the nucleus was stained with DAPI (blue). Scale bar 100 μm. (B) *mFam173b* mRNA expression after intrathecal *mFam173b*-*AS* injections in vehicle (*n* = 5 mice)- and CFA (*n* = 6 mice)-treated mice. (C–D) Time course of (C) thermal and (D) mechanical hyperalgesia following intraplantar injection of CFA (*n* = 8 mice) or vehicle (*n* = 4 mice), before and after intrathecal *mFam173b-AS* (set 2) or mismatch antisense ODN (MM-ODN) injections. (E, F) Intraplantar and intrathecal HSV amplicons encoding for hFAM173B and GFP (green) target sensory neurons. Successful sensory neuron expression of (E) GFP and (F) hFAM173B was observed in the DRG but not in other cells in the DRG such as F4/80-positive macrophages. Nuclei are visualized with DAPI, scale bar 20 μm. (G) Intraplantar HSV-hFAM173B induces expression of hFAM173B in the DRG but not in SC. Black line is 25-kDa marker. (H–I) Expression of GFP, as indicator of successful transgene expression, was observed in (H) peripherin-positive sciatic nerve fibers (scale bar 20 μm) and (I) peripherin-positive nerve endings in the plantar skin of the hind paw (scale bar 25 μm) at 2 days after the last intraplantar HSV-FAM173B injection. (J–K) Intrathecal HSV-hFAM173B injections rescued *mFam173b-AS*–mediated (set 1) attenuation of CFA-induced (J) thermal and (K) mechanical hyperalgesia (*n* = 8 mice). (L–M) Intrathecal HSV-hFAM173B prolonged carrageenan-induced (*n* = 4–12 mice) transient inflammatory (L) thermal and (M) mechanical hypersensitivity. (N) *mFam173b* mRNA expression in DRGs after intraplantar CFA (*n* = 9 mice) at day 1, 3, and 7. Data are represented as mean ± SEM. * = *P* < 0.05; ** = *P* < 0.01; *** = *P* < 0.001. Statistical analyses were performed by unpaired two-tailed *t* tests (B), by one-way ANOVA (N), or by two-way repeated measures ANOVA ([C, D], [J–M]) with Holm-Sidak multiple comparison test. For Fig 1L/1M, we used a two-way ANOVA with Holm-Sidak multiple comparison test because we combined several experiments and did not have equal numbers to perform a two-way repeated measures ANOVA. Underlying data can be found in S1 Data. CFA, complete Freund’s adjuvant; DAPI, 4′,6-diamidino-2-phenylindole; DRG, dorsal root ganglia; EV, empty vector; GFAP, glial fibrillary acidic protein; GFP, green fluorescent protein; HSV, herpes simplex virus; Iba1, ionized calcium binding adaptor molecule 1; MM-ODN, mismatch ODN; NF200, neurofilament 200; ODN, oligodeoxynucleotide; SC, spinal cord; SEM, standard error of the mean.
(TIF)

S2 Fig FAM173B is a mitochondrial lysine-specific methyltransferase. (A) *hFAM173B* and (B) *mFam173b* mRNA are expressed in all tissues examined. β-actin and HPRT mRNAs are shown as controls. (C) Full-length hFAM173B methylates lysine-homopolymers (*n* = 3 MTase reactions). (D) HSV-mediated expression of WT hFAM173B or hFAM173B-D94A induced similar expression levels in N2A cells. (E) *mFam173b-GFP* and hFAM173B-D94A-GFP colocalize with the mitochondrial dye MitoTrackerRedCMXROS. Scale bar 10 μm. (F) Western blot analyses of mitochondrial (M) and cytosolic (C) fraction of N2A cells overexpressing control (EV) and hFAM173B. COXIV and β-tubulin were used as mitochondrial and cytosolic loading marker, respectively. (G) Western blot analysis of WT hFAM173B and the methyltransferase-deficient mutant hFAM173B-D94A indicate that both are expressed in mitochondria. (H) Electron microscopy of immunogold labeling of GFP (left panel) or GFP-tagged hFAM173B (right panel) in N2A cells. Data are represented as mean ± SEM. * = *P* < 0.05. A statistical analysis was performed by a one-way ANOVA with Holm-Sidak multiple comparison test (C). Underlying data can be found in S1 Data. COXIV, cytochrome c oxidase IV; EV, empty vector; GFP, green fluorescent protein; HPRT, Hypoxanthine Phosphoribosyltransferase 1; HSV, herpes simplex virus; M, mitochondrion; MTase, methyltransferase; N2A, Neuro2a; SEM, standard error of the mean; WT, wild-type.
(TIF)

S3 Fig FAM173B influences mitochondrial potential and promotes superoxides and ROS. (A) *mFam173b-AS* efficiently reduced *mFam173b* mRNA expression in N2A cells (*n* = 6 wells). (B–C) Exemplar images of MitoTrackerRedCMXROS staining after (B) *mFam173b-AS*–mediated knockdown of mFam173b or (C) overexpression of hFAM173B. Scale bar 10 μm. (D) HSV-mediated hFAM173B overexpression in N2A cells is detected by western blot. (E) HSV amplicons encoding for hFAM173B-GFP selectively target sensory neurons in vitro. Nuclei are visualized with DAPI. Scale bar 50 μm. (F) ΔTMRM fluorescence 48 hours after hFAM173B overexpression in N2A cells (*n* = 97–110 cells). (G, H) hFAM173B overexpression in (G) N2A (*n* = 10 wells) and (H) HEK293 (*n* = 9 wells) cells increased DHE fluorescence. (I) hFAM173B overexpression in N2A cells increased MitoSox fluorescence compared to controls (EV) (*n* = 8 wells). (J) MitoTrackerRedCMH_2_-XROS fluorescence intensity at day 3 (*n* = 9 mice) and day 6 (EV *n* = 4; hFAM173B *n* = 6 mice) in medium- and/or large-diameter neurons after intraplantar carrageenan injection. (K) Exemplar images of quantified MitoTrackerRedCM-H_2_XROS fluorescence at day 3 after carrageenan. Scale bar 50 μm. Data are represented as mean ± SEM. ** = *P* < 0.01; *** = *P* < 0.001. Statistical analyses were performed by unpaired two-tailed *t* tests (A, F, H–K). Underlying data can be found in S1 Data. DAPI, 4′,6-diamidino-2-phenylindole; DHE, dihydroethidium; EV, empty vector; HEK293, human embryonic kidney 293 cells; HSV, herpes simplex virus; MM-ODN, mismatch ODN; N2A, Neuro2a; ODN, oligodeoxynucleotide; ROS, reactive oxygen species; SEM, standard error of the mean; TMRM, tetramethylrhodamine methyl ester.
(TIF)

S4 Fig FAM173B promotes microglia/macrophage activation via an ROS-dependent pathway. (A) Increased spinal microglia TNFα release after stimulation with supernatants of TNFα-stimulated sensory neurons overexpressing hFAM173B (EV *n* = 20; hFAM173B *n* = 30 wells; 100% = 28 pg/ml based on the mean of 3 independent experiments). (B) Anti-TNFα neutralizing antibody (HSV-FAM173B: *n* = 6; HSV-EV: *n* = 4 mice) and (C) minocycline (minocycline: *n* = 12; vehicle: *n* = 6 mice) attenuated the hFAM173B-mediated prolongation of carrageenan-induced thermal hypersensitivity. (D) Example of spinal cord and the areas used for quantification (light grey) of Iba1 immunofluorescence. (E) Quantification of Iba1 expression in the dorsal horn of the spinal cord of mice with or without sensory neuron overexpression of hFAM173B at 1 month after carrageenan and 24 h after inhibition of ROS using intraperitoneal PBN injections (EV *n* = 7; hFAM173B *n* = 5 mice). (F–G) *mFam173b-AS* treatment to knock down mFam173b did not affect *GFAP* mRNA expression in (F) spinal cord and (G) DRG in the CFA model of persistent inflammatory pain (*n* = 8 mice). Data are represented as mean ± SEM. *** = *P* < 0.001. Statistical analyses were performed by unpaired two-tailed *t* tests (A, E–G) or by two-way repeated measures ANOVA (B, C) with Holm-Sidak multiple comparison test. Underlying data can be found in S1 Data. CFA, complete Freund’s adjuvant; DRG, dorsal root ganglia; EV, empty vector; HSV, herpes simplex virus; Iba1, ionized calcium binding adaptor molecule 1; PBN, phenyl-N-*t*-butylnitrone; ROS, reactive oxygen species; SEM, standard error of the mean; TNFα, tumor necrosis factor α; veh, vehicle.
(TIF)

S5 Fig FAM173B methyltransferase activity is required for development of persistent pain. Time course of (A) thermal and (B) mechanical hypersensitivity following intraplantar carrageenan injection in mice receiving intraplantar HSV-hFAM173B, HSV-hFAM173B-D94A, or HSV-EV injections (EV *n* = 10; hFAM173B and hFAM173B-D94A *n* = 8 mice). (C) Exemplar images of quantified Iba1 staining in Fig 8C/8D. Scale bar 100 μm for spinal cord and 50 μm for DRG. (D) Exemplar images of quantified DHE staining in Fig 8F. Scale bar 20 μm. (E) Supernatants of TNFα-stimulated sensory neurons overexpressing hFAM173B-D94A did not increase TNFα release by spinal microglia in vitro to the same extent as supernatant of sensory neurons expression the WT hFAM173B (hFAM173b *n* = 30; hFAM173B-D94A *n* = 20 wells). Data are represented as mean ± SEM. * = *P* < 0.05; *** = *P* < 0.001. Statistical analyses were performed by an unpaired two-tailed *t* test (E) or by two-way repeated measures ANOVA (A, B) with Holm-Sidak multiple comparison test. Underlying data can be found in S1 Data. DHE, dihydroethidium; DRG, dorsal root ganglia; EV, empty vector; Iba1, ionized calcium binding adaptor molecule 1; SEM, standard error of the mean; TNFα, tumor necrosis factor α; WT, wild-type.
(TIF)

## Abbreviations

7BS: 7 β-strands
ATP: adenosine triphosphate
BDNF: Brain-derived neurotrophic factor
CCL2: chemokine (C-C motif) ligand 2
CCT5: Chaperonin Containing TCP1 Subunit 5
CFA: complete Freund’s adjuvant
COMT: catechol-O-methyltransferase
contra: contralateral
COXIV: cytochrome c oxidase IV
CPP: conditioned place preference
DAPI: 4′,6-diamidino-2-phenylindole
DHE: dihydroethidium
DRG: dorsal root ganglia
DWB: dynamic weight bearing
EV: empty vector
FCCP: carbonyl cyanide p-trifluoromethoxyphenylhydrazone
GAPDH: glyceraldehyde 3-phosphate dehydrogenase
GFAP: glial fibrillary acidic protein
GFP: green fluorescent protein
GWAS: genome-wide association study
HEK293: human embryonic kidney 293 cells
HPRT: Hypoxanthine Phosphoribosyltransferase 1
HSV: herpes simplex virus
Iba1: ionized calcium binding adaptor molecule 1
IgG: immunoglobulin
IL6: interleukin 6
ipsi: ipsilateral
M: mitochondrion
MCP1: monocyte chemoattractant protein 1
MM-ODN: mismatch ODN
MTase: methyltransferase
N: nucleus
N2A: Neuro2a
ODN: oligodeoxynucleotide
PBN: phenyl-N-*t*-butylnitrone
PDI: protein disulfide-isomerase
ROS: reactive oxygen species
SAM: *S*-adenosyl-L-methionine
SC: spinal cord
SEM: standard error of the mean
SNI: spared nerve injury
SNP: single nucleotide polymorphism
TMRM: tetramethylrhodamine methyl ester
TNFα: tumor necrosis factor α
veh: vehicle
WT: wild-type
ΔΨm: mitochondrial membrane potential
