# Supplementary figures and images for: Identification of FAM173B as a protein methyltransferase promoting chronic pain
Source: PLoS Biol. 2018 Feb 14;16(2):e2003452. doi: 10.1371/journal.pbio.2003452 (PMC5828452; doi:10.1371/journal.pbio.2003452)

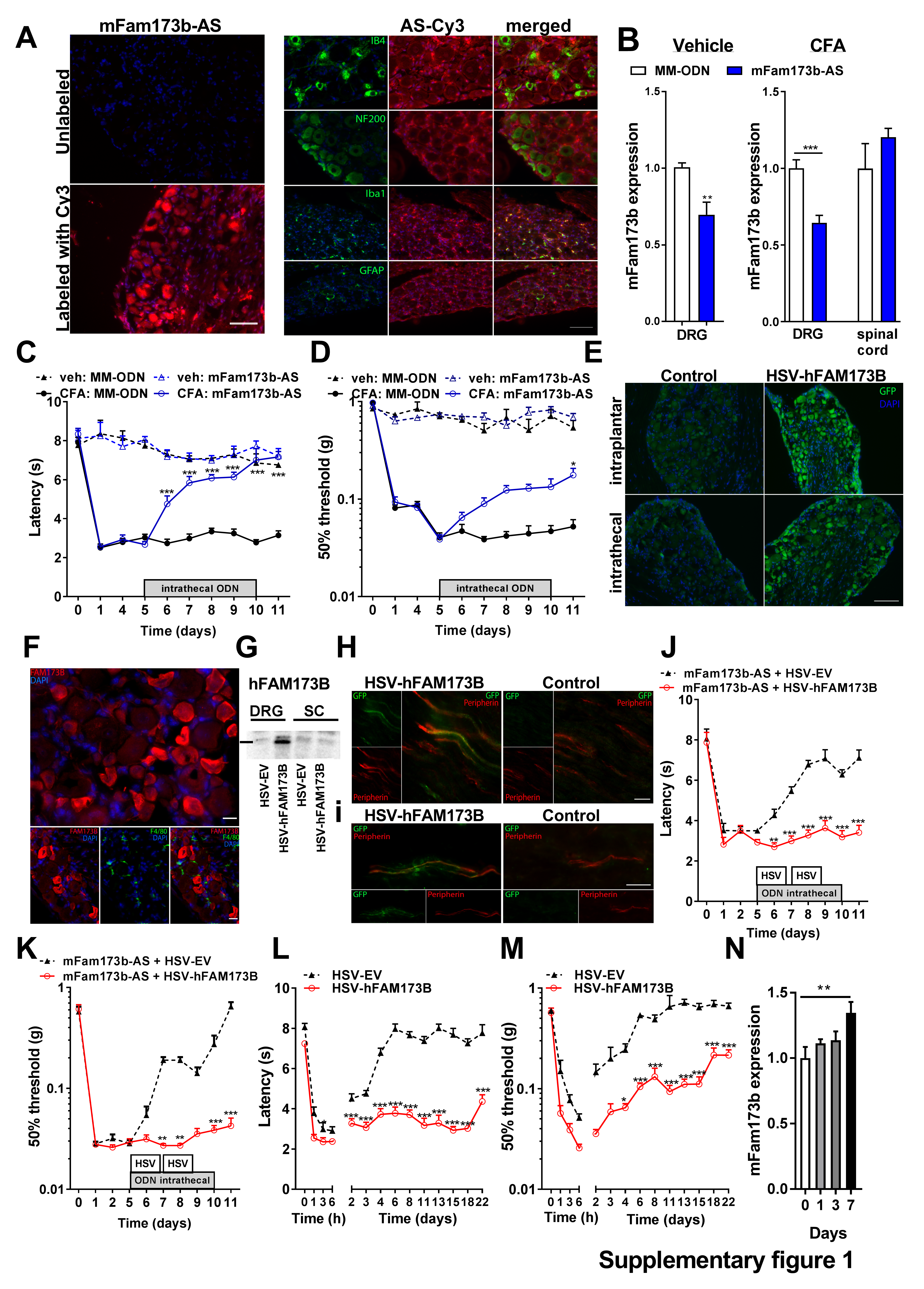

Supplement: S1 Fig — (A) Left images represent DRG of mice injected with either labeled (Cy3, red) or unlabeled mouse Fam173b antisense ODNs (mFam173b-AS). Labeling is visible in sensory neurons as well as some cells surrounding the sensory neurons. Scale bar 50 μm. Right images: After intrathecal Cy3-labeled mFam173b-AS injections (red), lumbar DRG from mice were stained for IB4, NF200, Iba1, and GFAP (green), and the nucleus was stained with DAPI (blue). Scale bar 100 μm. (B) mFam173b mRNA expression after intrathecal mFam173b-AS injections in vehicle (n = 5 mice)- and CFA (n = 6 mice)-treated mice. (C–D) Time course of (C) thermal and (D) mechanical hyperalgesia following intraplantar injection of CFA (n = 8 mice) or vehicle (n = 4 mice), before and after intrathecal mFam173b-AS (set 2) or mismatch antisense ODN (MM-ODN) injections. (E, F) Intraplantar and intrathecal HSV amplicons encoding for hFAM173B and GFP (green) target sensory neurons. Successful sensory neuron expression of (E) GFP and (F) hFAM173B was observed in the DRG but not in other cells in the DRG such as F4/80-positive macrophages. Nuclei are visualized with DAPI, scale bar 20 μm. (G) Intraplantar HSV-hFAM173B induces expression of hFAM173B in the DRG but not in SC. Black line is 25-kDa marker. (H–I) Expression of GFP, as indicator of successful transgene expression, was observed in (H) peripherin-positive sciatic nerve fibers (scale bar 20 μm) and (I) peripherin-positive nerve endings in the plantar skin of the hind paw (scale bar 25 μm) at 2 days after the last intraplantar HSV-FAM173B injection. (J–K) Intrathecal HSV-hFAM173B injections rescued mFam173b-AS–mediated (set 1) attenuation of CFA-induced (J) thermal and (K) mechanical hyperalgesia (n = 8 mice). (L–M) Intrathecal HSV-hFAM173B prolonged carrageenan-induced (n = 4–12 mice) transient inflammatory (L) thermal and (M) mechanical hypersensitivity. (N) mFam173b mRNA expression in DRGs after intraplantar CFA (n = 9 mice) at day 1, 3, and 7. Data are represented [file pbio.2003452.s002.tif]

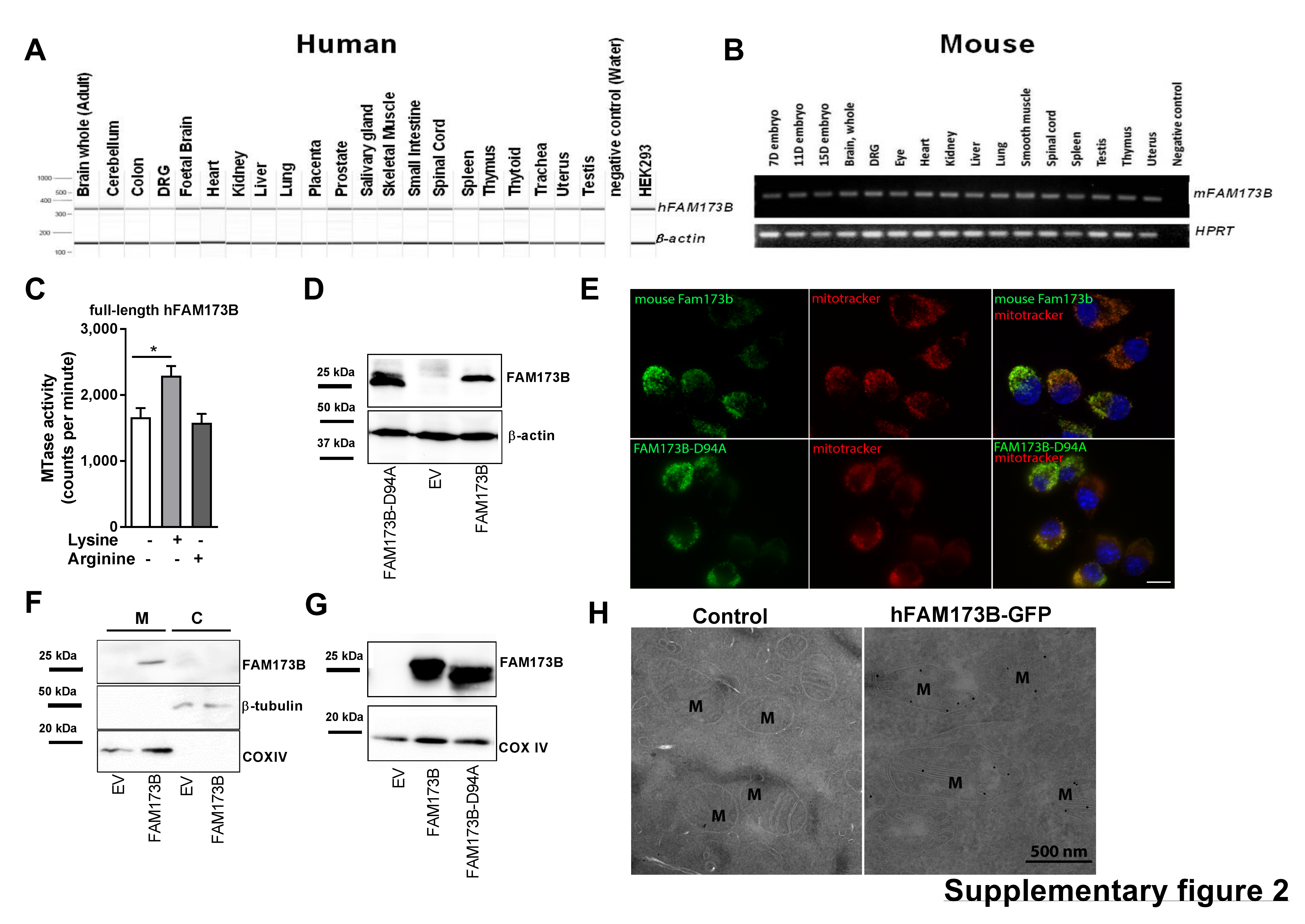

Supplement: S2 Fig — (A) hFAM173B and (B) mFam173b mRNA are expressed in all tissues examined. β-actin and HPRT mRNAs are shown as controls. (C) Full-length hFAM173B methylates lysine-homopolymers (n = 3 MTase reactions). (D) HSV-mediated expression of WT hFAM173B or hFAM173B-D94A induced similar expression levels in N2A cells. (E) mFam173b-GFP and hFAM173B-D94A-GFP colocalize with the mitochondrial dye MitoTrackerRedCMXROS. Scale bar 10 μm. (F) Western blot analyses of mitochondrial (M) and cytosolic (C) fraction of N2A cells overexpressing control (EV) and hFAM173B. COXIV and β-tubulin were used as mitochondrial and cytosolic loading marker, respectively. (G) Western blot analysis of WT hFAM173B and the methyltransferase-deficient mutant hFAM173B-D94A indicate that both are expressed in mitochondria. (H) Electron microscopy of immunogold labeling of GFP (left panel) or GFP-tagged hFAM173B (right panel) in N2A cells. Data are represented as mean ± SEM. * = P < 0.05. A statistical analysis was performed by a one-way ANOVA with Holm-Sidak multiple comparison test (C). Underlying data can be found in S1 Data. COXIV, cytochrome c oxidase IV; EV, empty vector; GFP, green fluorescent protein; HPRT, Hypoxanthine Phosphoribosyltransferase 1; HSV, herpes simplex virus; M, mitochondrion; MTase, methyltransferase; N2A, Neuro2a; SEM, standard error of the mean; WT, wild-type. (TIF) [file pbio.2003452.s003.tif]

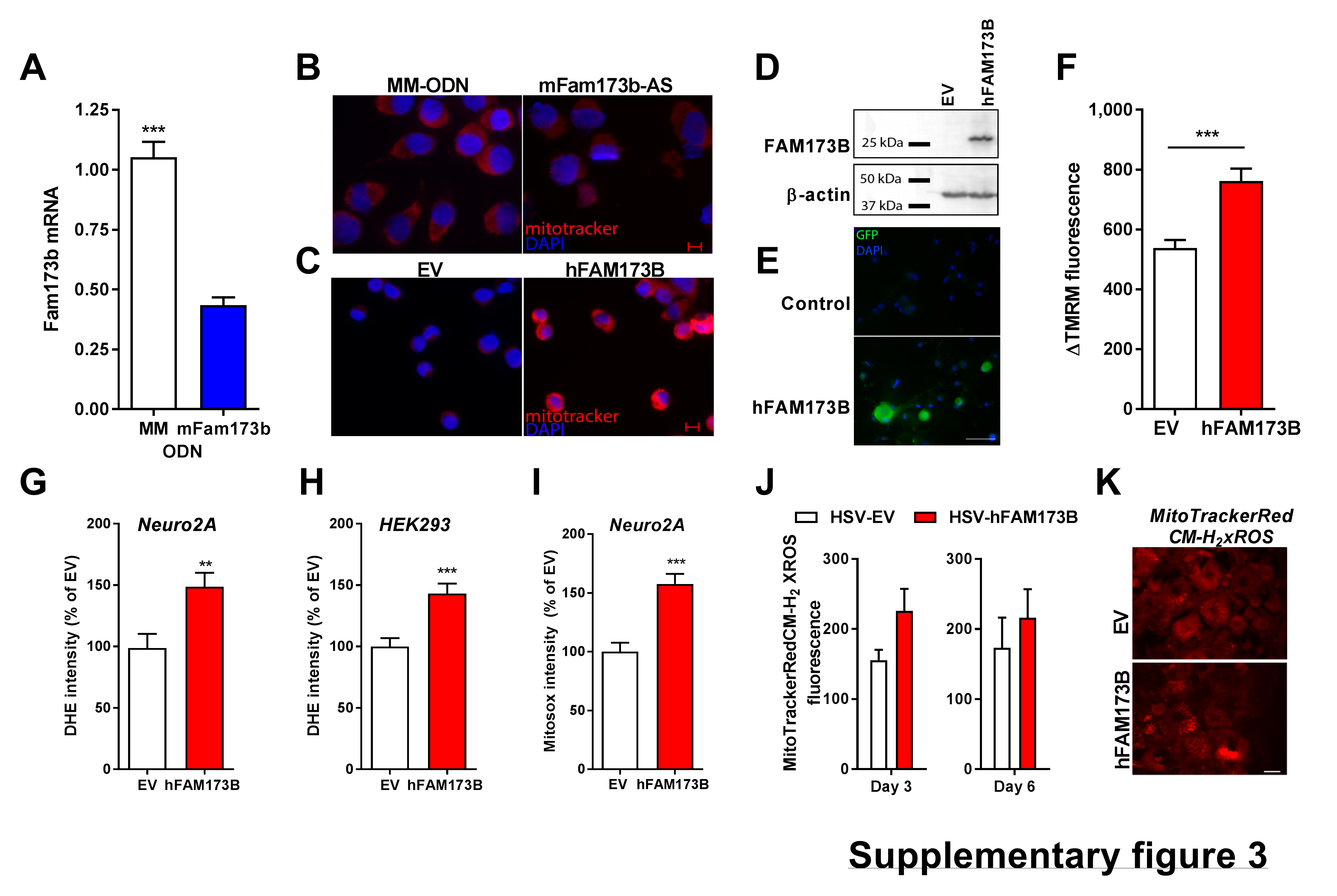

Supplement: S3 Fig — (A) mFam173b-AS efficiently reduced mFam173b mRNA expression in N2A cells (n = 6 wells). (B–C) Exemplar images of MitoTrackerRedCMXROS staining after (B) mFam173b-AS–mediated knockdown of mFam173b or (C) overexpression of hFAM173B. Scale bar 10 μm. (D) HSV-mediated hFAM173B overexpression in N2A cells is detected by western blot. (E) HSV amplicons encoding for hFAM173B-GFP selectively target sensory neurons in vitro. Nuclei are visualized with DAPI. Scale bar 50 μm. (F) ΔTMRM fluorescence 48 hours after hFAM173B overexpression in N2A cells (n = 97–110 cells). (G, H) hFAM173B overexpression in (G) N2A (n = 10 wells) and (H) HEK293 (n = 9 wells) cells increased DHE fluorescence. (I) hFAM173B overexpression in N2A cells increased MitoSox fluorescence compared to controls (EV) (n = 8 wells). (J) MitoTrackerRedCMH2-XROS fluorescence intensity at day 3 (n = 9 mice) and day 6 (EV n = 4; hFAM173B n = 6 mice) in medium- and/or large-diameter neurons after intraplantar carrageenan injection. (K) Exemplar images of quantified MitoTrackerRedCM-H2XROS fluorescence at day 3 after carrageenan. Scale bar 50 μm. Data are represented as mean ± SEM. ** = P < 0.01; *** = P < 0.001. Statistical analyses were performed by unpaired two-tailed t tests (A, F, H–K). Underlying data can be found in S1 Data. DAPI, 4′,6-diamidino-2-phenylindole; DHE, dihydroethidium; EV, empty vector; HEK293, human embryonic kidney 293 cells; HSV, herpes simplex virus; MM-ODN, mismatch ODN; N2A, Neuro2a; ODN, oligodeoxynucleotide; ROS, reactive oxygen species; SEM, standard error of the mean; TMRM, tetramethylrhodamine methyl ester. (TIF) [file pbio.2003452.s004.tif]

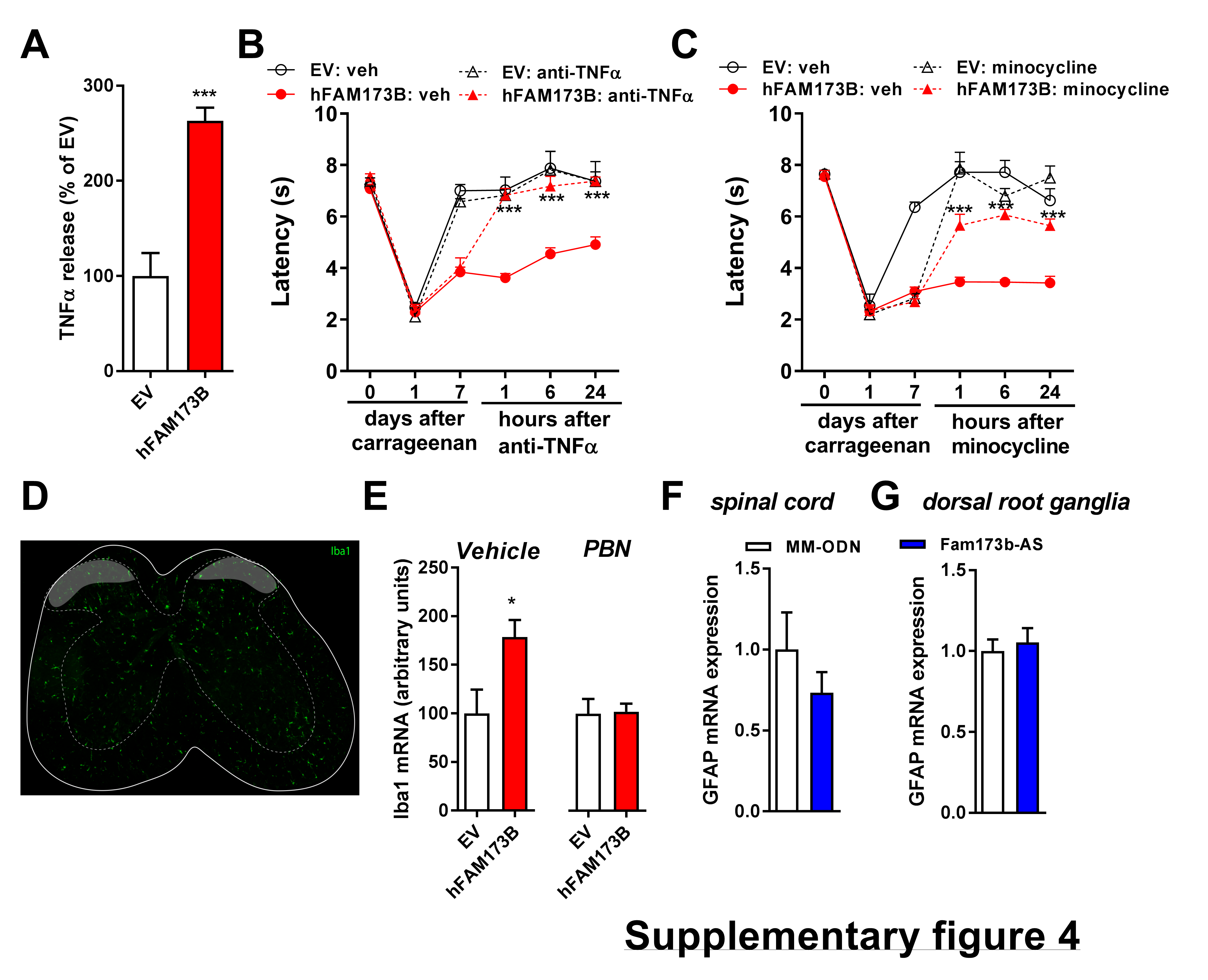

Supplement: S4 Fig — (A) Increased spinal microglia TNFα release after stimulation with supernatants of TNFα-stimulated sensory neurons overexpressing hFAM173B (EV n = 20; hFAM173B n = 30 wells; 100% = 28 pg/ml based on the mean of 3 independent experiments). (B) Anti-TNFα neutralizing antibody (HSV-FAM173B: n = 6; HSV-EV: n = 4 mice) and (C) minocycline (minocycline: n = 12; vehicle: n = 6 mice) attenuated the hFAM173B-mediated prolongation of carrageenan-induced thermal hypersensitivity. (D) Example of spinal cord and the areas used for quantification (light grey) of Iba1 immunofluorescence. (E) Quantification of Iba1 expression in the dorsal horn of the spinal cord of mice with or without sensory neuron overexpression of hFAM173B at 1 month after carrageenan and 24 h after inhibition of ROS using intraperitoneal PBN injections (EV n = 7; hFAM173B n = 5 mice). (F–G) mFam173b-AS treatment to knock down mFam173b did not affect GFAP mRNA expression in (F) spinal cord and (G) DRG in the CFA model of persistent inflammatory pain (n = 8 mice). Data are represented as mean ± SEM. *** = P < 0.001. Statistical analyses were performed by unpaired two-tailed t tests (A, E–G) or by two-way repeated measures ANOVA (B, C) with Holm-Sidak multiple comparison test. Underlying data can be found in S1 Data. CFA, complete Freund’s adjuvant; DRG, dorsal root ganglia; EV, empty vector; HSV, herpes simplex virus; Iba1, ionized calcium binding adaptor molecule 1; PBN, phenyl-N-t-butylnitrone; ROS, reactive oxygen species; SEM, standard error of the mean; TNFα, tumor necrosis factor α; veh, vehicle. (TIF) [file pbio.2003452.s005.tif]

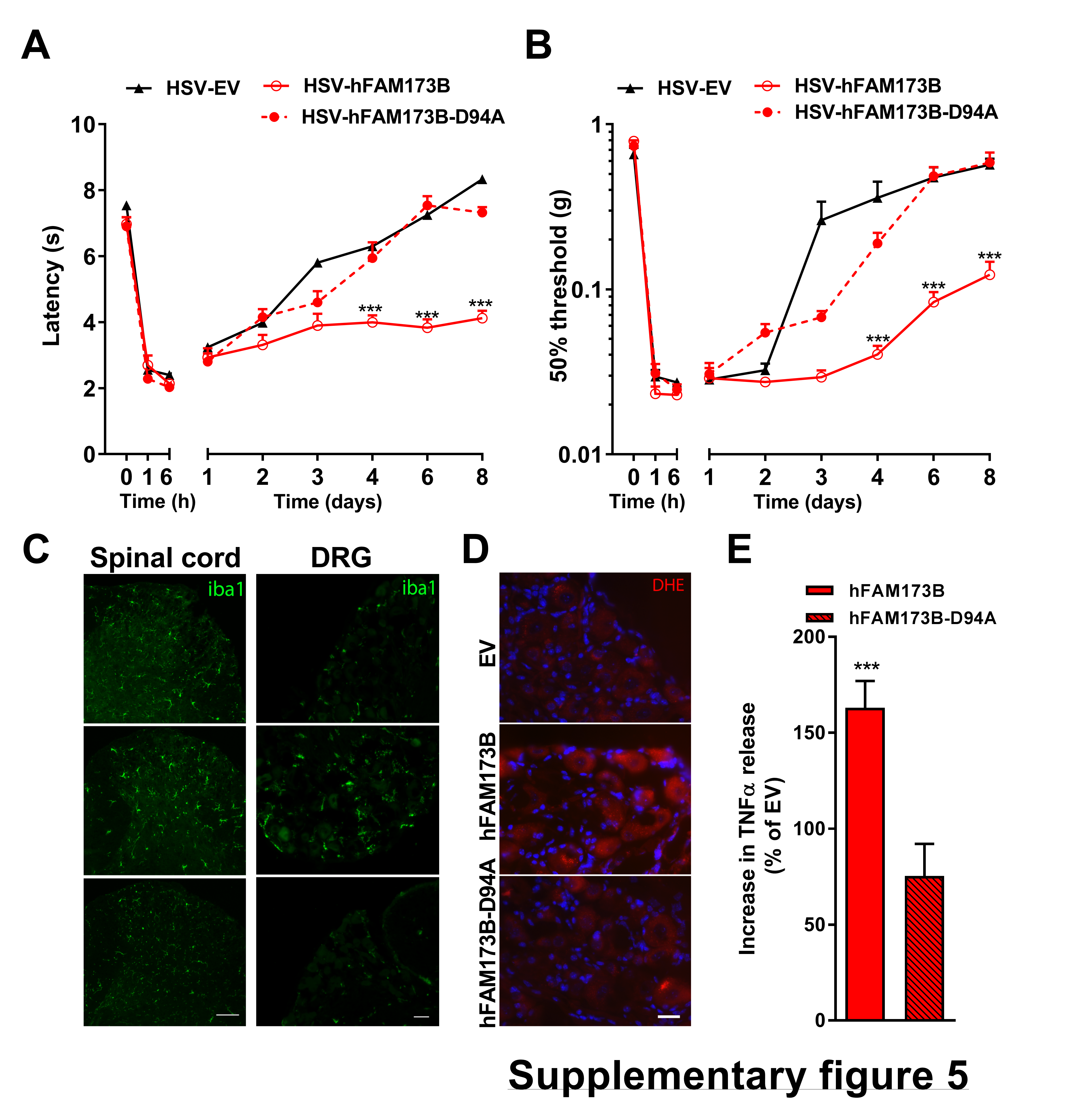

Supplement: S5 Fig — Time course of (A) thermal and (B) mechanical hypersensitivity following intraplantar carrageenan injection in mice receiving intraplantar HSV-hFAM173B, HSV-hFAM173B-D94A, or HSV-EV injections (EV n = 10; hFAM173B and hFAM173B-D94A n = 8 mice). (C) Exemplar images of quantified Iba1 staining in Fig 8C/8D. Scale bar 100 μm for spinal cord and 50 μm for DRG. (D) Exemplar images of quantified DHE staining in Fig 8F. Scale bar 20 μm. (E) Supernatants of TNFα-stimulated sensory neurons overexpressing hFAM173B-D94A did not increase TNFα release by spinal microglia in vitro to the same extent as supernatant of sensory neurons expression the WT hFAM173B (hFAM173b n = 30; hFAM173B-D94A n = 20 wells). Data are represented as mean ± SEM. * = P < 0.05; *** = P < 0.001. Statistical analyses were performed by an unpaired two-tailed t test (E) or by two-way repeated measures ANOVA (A, B) with Holm-Sidak multiple comparison test. Underlying data can be found in S1 Data. DHE, dihydroethidium; DRG, dorsal root ganglia; EV, empty vector; Iba1, ionized calcium binding adaptor molecule 1; SEM, standard error of the mean; TNFα, tumor necrosis factor α; WT, wild-type. (TIF) [file pbio.2003452.s006.tif]
